# A pipeline for targeted metagenomics of environmental bacteria

**DOI:** 10.1186/s40168-020-0790-7

**Published:** 2020-02-15

**Authors:** Anissa Grieb, Robert M. Bowers, Monike Oggerin, Danielle Goudeau, Janey Lee, Rex R. Malmstrom, Tanja Woyke, Bernhard M. Fuchs

**Affiliations:** 1 Max Planck Institute for Marine Microbiology, Celsiusstraße 1, 28359 Bremen, Germany; 2 DOE Joint Genome Institute, Lawrence Berkeley National Laboratory, Mail Stop: 91R183, 1 Cyclotron Road, Berkeley, CA 94720 USA

**Keywords:** FACS, Cell fixation, HCR-FISH, Mini-metagenomics

## Abstract

**Background:**

Metagenomics and single cell genomics provide a window into the genetic repertoire of yet uncultivated microorganisms, but both methods are usually taxonomically untargeted. The combination of fluorescence in situ hybridization (FISH) and fluorescence activated cell sorting (FACS) has the potential to enrich taxonomically well-defined clades for genomic analyses.

**Methods:**

Cells hybridized with a taxon-specific FISH probe are enriched based on their fluorescence signal via flow cytometric cell sorting. A recently developed FISH procedure, the hybridization chain reaction (HCR)-FISH, provides the high signal intensities required for flow cytometric sorting while maintaining the integrity of the cellular DNA for subsequent genome sequencing. Sorted cells are subjected to shotgun sequencing, resulting in targeted metagenomes of low diversity.

**Results:**

Pure cultures of different taxonomic groups were used to (1) adapt and optimize the HCR-FISH protocol and (2) assess the effects of various cell fixation methods on both the signal intensity for cell sorting and the quality of subsequent genome amplification and sequencing. Best results were obtained for ethanol-fixed cells in terms of both HCR-FISH signal intensity and genome assembly quality. Our newly developed pipeline was successfully applied to a marine plankton sample from the North Sea yielding good quality metagenome assembled genomes from a yet uncultivated flavobacterial clade.

**Conclusions:**

With the developed pipeline, targeted metagenomes at various taxonomic levels can be efficiently retrieved from environmental samples. The resulting metagenome assembled genomes allow for the description of yet uncharacterized microbial clades.

Video abstract.

## Background

Shotgun metagenomics has become standard in microbial ecology studies owing to increases in sequencing throughput at dropping cost and continued improvements in bioinformatic analysis pipelines. As little as 1 pg of DNA is sufficient to provide ecological insights into a given microbial community [1, 2]. Current bioinformatic analyses focus on the assembly of large contigs from metagenomic reads and the annotation of genes in order to reveal the metabolic potential of the sequenced community members. The sequencing depth of metagenomes exceeding hundreds of millions of reads allows for the assembly and binning of nearly complete genomes (metagenome-assembled genomes, MAGs [3]) and hence facilitates the prediction of physiological traits of individual species within microbial communities. However, linking metabolic traits to specific organisms can still not yet be realized for many environmental taxa of interest due to missing phylogenetic markers such as 16S rRNA gene sequences in the MAGs [4, 5]. Single cell genomics (SCG) combines the taxonomic classification from the 16S rRNA gene of a single cell with the genomic information including functional annotation [6]. In this approach, individual cells are separated, e.g., by fluorescence activated cell sorting (FACS) of DNA-stained cells, and subjected to multiple displacement amplification (MDA)-mediated whole genome amplification and sequencing. The resulting single amplified genomes (SAGs) are generally incomplete [7, 8]. Both metagenomics and SCG are usually untargeted and this random selection might challenge access to rare organisms [9, 10]. These could be recovered by a targeted approach where specific microbial groups are enriched prior to sequencing.

Fluorescence in situ hybridization (FISH) using fluorescently labeled oligonucleotide probes provide such a targeted approach by staining specifically the microorganism of interest before enriching them by fluorescence activated cell sorting (FACS). As the specificity of the 16S rRNA-targeted probes can be designed for different taxonomic levels from domain down to sub-genus level, flow cytometric sorting of stained cells yields taxonomically well-defined cell enrichments in high purity. Sorting of FISH-stained cells has previously been done in several studies using either fluorescently labeled oligonucleotide probes [11–13] or probes labeled with horseradish-peroxidase that catalyzes the deposition of fluorescently labeled tyramides (CARD-FISH) [14]. These studies have sequenced PCR products of specific genes like the 16S rRNA from sorted cells. Whole genome sequencing has been attempted from FISH labeled and sorted cells, but the recovered genomes suffered from low completeness [15, 16]. Developing new FISH protocols, optimized for genome recovery after FACS are necessary for the targeted sequencing of specific taxonomic clades.

A key prerequisite for a targeted FISH&FACS mini-metagenomic approach is a strong fluorescence signal for flow cytometric cell sorting. FISH with direct fluorescently labeled probes often shows too low signal-to-noise ratios for small cells from oligotrophic environments to be detected by flow cytometers [17]. CARD-FISH [18] provides signals that are 26 to 41-fold brighter than FISH with mono-labeled probes [19], but involves a radical reaction with hydrogen peroxide which can damage the cellular DNA [20]. A radical-free alternative to CARD-FISH is the two-step hybridization chain reaction (HCR)-FISH which was applied to bacteria by Yamaguchi et al. [21]. In this approach, a specific oligonucleotide probe, carrying an initiator sequence, is hybridized to the cells. Next, two fluorescently labeled hairpin oligos (H1 and H2) bind subsequently in a chain reaction to the initiator sequence, thus multiplying the fluorescent signal. The detection rates with HCR-FISH were comparable to CARD-FISH for coastal picoplankton and sediment in epifluorescence microscopy [22]. For HCR-FISH, as opposed to CARD-FISH, cells do not need to be fixed with formaldehyde—a fixative that preserves the cell morphology, but impairs the DNA quality and thus downstream genome amplification [23].

In this study, we developed a combined HCR-FISH&FACS pipeline for the targeted retrieval of uncultivated bacterial clades from the environment. First, the impact of various cell fixation methods on the quality of whole genome amplification and assembly was tested using isolates. Next, the signals from HCR-FISH were improved by comparing different buffers and by introducing a denaturation step. The optimized protocol was validated on a set of isolates with various GC-contents. Finally, the pipeline was successfully applied on an environmental seawater sample for the targeted retrieval of the yet uncharacterized flavobacterial clade Vis6. Vis6 is one of several flavobacterial clades, which respond in tight succession to marine diatom blooms in spring around the island of Helgoland in the North Sea [24]. For many of these clades the metabolic capacities have recently been described based on cultured representatives and metagenome assembled genomes (MAGs) [25], but Vis6 has evaded cultivation so far and MAG affiliation was uncertain [26].

## Results

### Quantification of the HCR-FISH signal intensity depending on cell fixation

**Fig. 1:**
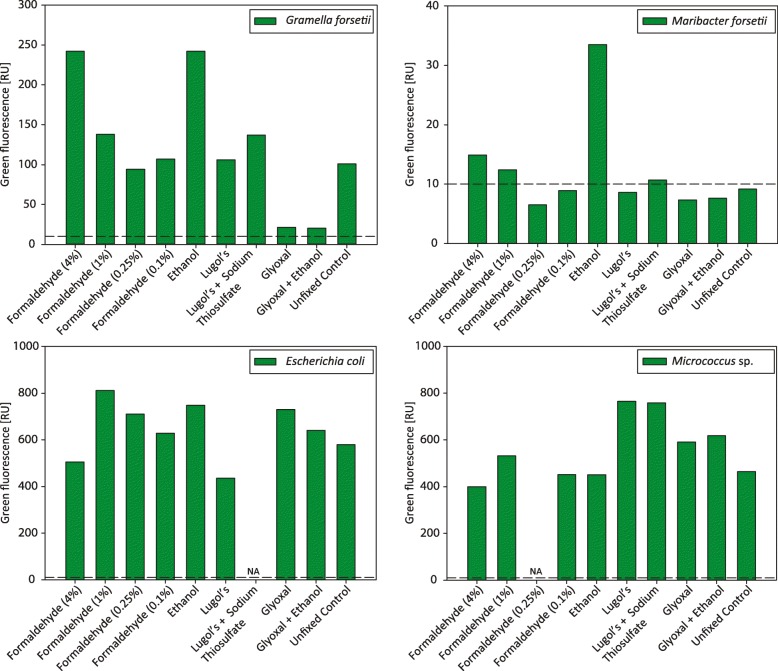
HCR-FISH fluorescence (green fluorescence, 530/40 nm band-pass filter) of four isolates treated with different fixatives. The median signal of the population from flow cytometric analysis is shown. The dashed line indicates the level of background noise. NA = not analyzed

We tested nine different fixation methods on four different bacterial strains to determine the impact of fixation on the fluorescence labeling of the cells, and quantified the fluorescence intensities after HCR-FISH by flow cytometry (Fig. 1). An overview of the different experiments leading to the final optimized protocol is shown in Additional file 1: Figure S1. *Maribacter forsetii* and *Gramella forsetii* are flavobacterial species, commonly found in planktonic seawater samples of the North Sea. With *Escherichia coli* we selected a *Gammaproteobacterium* and with *Micrococcus* sp. a gram-positive species to have a broader target group for optimization.

Generally, most of the different fixatives tested produced HCR-FISH signals well above background levels (at 10 RU, see Additional file 2: Figure S2) for *Escherichia coli*, for *Micrococcus* sp. and for *Gramella forsetii* cells except for both glyoxal fixations. In contrast, *Maribacter forsetii* showed detectable HCR-FISH signals for only formaldehyde and the ethanol fixation (Additional file 3: Figure S3).

Based on the flow cytometric intensity measurements, we chose three fixation methods for testing the downstream MDA amplification and sequencing quality after sorting FISH-positive cells (see Additional file 1: Figure S1 for an overview). All formaldehyde fixations with 4% and 1% final concentration yielded high signal intensities with HCR-FISH and showed a distinct population by flow cytometry (Additional file 3: Figure S3). Similarly, the brightest DNA staining with 4’,6-diamidino-2-phenylindole (DAPI) was achieved in formaldehyde-fixed cells (Additional file 4: Figure S4). However, as 4% formaldehyde fixation has previously been described to render the genomic DNA unusable for genome amplification and sequencing [23], only 1% formaldehyde fixation was chosen for further testing. Cell fixations with final concentrations of 0.25% and 0.1% formaldehyde were not further analyzed since hybridized cells showed low signal intensities and deteriorated morphologies (Additional file 3: Figure S3). The second method evaluated was ethanol fixation, which resulted in high signal intensities for all strains after HCR-FISH (Figure 1). For *Gramella forsetii* and *Maribacter forsetii* the signal intensities of ethanol-fixed cells were even higher than for cells fixed with 1% formaldehyde. The third fixation method tested was based on Lugol’s solution. This method showed different results for each strain. While a distinct population for *Gramella forsetii* and *Micrococcus* sp. was discernible by flow cytometry, only low signal intensities were observed for *Maribacter forsetii*. *Escherichia coli* had an additional unstained population below the background level next to well-stained cells (Additional file 3: Figure S3). Lugol’s fixation in combination with a thiosulfate destaining resulted in heavily compromised *Escherichia coli* cells, which could not be further analyzed by flow cytometry. Glyoxal fixation was not analyzed further as it resulted generally in low signal intensities for all tested strains. Additionally, due to its cross-linking behavior comparable to formaldehyde [27], we expect detrimental effects on the DNA and thus low genome quality. Unfixed, hybridized cells served as controls. Their signal intensities were often lower than the other fixation methods and in case of *Gramella forsetii* showed signs of cell deterioration (Additional file 3: Figure S3 and Additional file 5: Figure S5).

### Impact of cell fixation on genome assembly

The next step in development of our HCR-FISH&FACS pipeline was the assessment of the MDA products and sequencing results of each strain after fixation, HCR-FISH and flow cytometric cell sorting. The genome quality of hybridized cells fixed with 1% formaldehyde, ethanol and Lugol’s solution was analyzed and compared to two controls using unfixed cells after HCR-FISH and unfixed, unhybridized cells. Additionally, we compared 100 vs. 500 sorted cells.

Formaldehyde fixed cells never yielded sufficient MDA product in any experiment (Additional file 6: Figure S6). For all other fixation methods tested, the crossing point in MDA amplification (CP; time of inflection point of real-time amplification curve) was reached on average 22% faster with 500 cells input compared to 100 cells input (Additional file 7: Figure S7). There were also no evident differences in CP values between the fixatives other than formaldehyde.

The amplified DNA from *Gramella forsetii* and *Maribacter forsetii* was sequenced to determine the impact of fixative on genome recovery. Using quality metrics of total assembly length, number of contigs, contig N50, and the numbers of misassemblies and mismatches, the quality of assemblies from 500 sorted cells was superior to assemblies derived from 100 sorted cells of the same batch (Additional file 8: Figure S8). Between the differently fixed and hybridized 500 cells batches, the differences in genome quality metrices were not significant, except of ethanol-fixed, hybridized cells having a higher N50 and a lower number of misassemblies compared to hybridized cells from unfixed samples. We analyzed the read coverages from fixed, hybridized, and sorted strains against the respective reference genomes (Additional file 9: Figure S9). Generally, the read coverages from 500 cells were higher than those from 100 cells for both strains, *Gramella forsetii* and *Maribacter forsetii*. For *Gramella forsetii* no difference could be detected in coverage between ethanol and Lugol’s fixation and unfixed cells, but the coverage for sorted *Maribacter forsetii* cells was clearly reduced for hybridized, unfixed cells and cells fixed with Lugol’s solution compared to ethanol fixed cells and the treatment control (unfixed and unhybridized).

### HCR-FISH optimization

For environmental samples, bright FISH signals are needed for detection by flow cytometry, because of the higher background noise, e.g., from particles, compared to cultures. In this study, we used HCR-FISH as a radical-free FISH signal amplification technique and further optimized the HCR-FISH protocol from Yamaguchi et al. [21] in several steps, including using H1/H2 amplifier hairpin probes containing four fluorochromes per probe compared to the two fluorochromes H1/H2 in the original protocol.

**Fig. 2:**
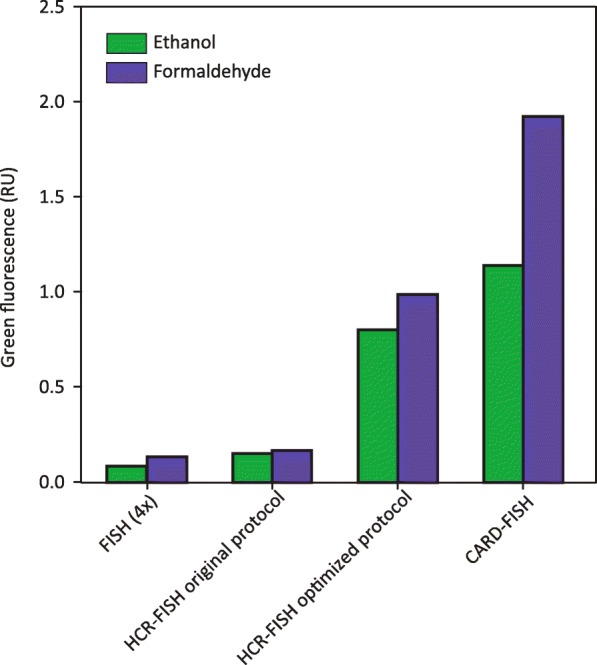
HCR-FISH signal intensity of a *Gramella forsetii* pure culture, fixed with formaldehyde (1%) or ethanol, as measured by microscopy. FISH with probes containing 4 fluorochromes was compared to two different HCR-FISH protocols (prior to and after optimization) and CARD-FISH. The optimized HCR-FISH protocol includes 30 min denaturation at 65°C prior to 2 h of hybridization with hybridization buffer B and 120 min of amplification. The original protocol did not include a denaturation step and 2 h of hybridization with hybridization buffer A were followed by 45 min of amplification

Altogether, the greatest improvements to HCR-FISH fluorescence signals on 1% formaldehyde fixed cells resulted from adding a denaturation step at 85°C prior to hybridization, switching from buffer A to buffer B for hybridization, and increasing the chain reaction amplification times to 120 min, which resulted in 69% of the CARD-FISH signal (Additional file 10: Figure S10). Buffer B contained more crowding reagents (blocking reagent, SDS, dextrane sulfate, and salmon sperm) compared to buffer A, boosting the efficiency of hybridization. The greatest increase in signal intensity was observed by introducing a denaturation step prior to hybridization. This likely linearizes the long probe with attached linker to resolve potential secondary structures in the ribosomal target region. With ethanol fixed cells, the signals increased from 45 °C to 75 °C denaturation (e.g., from 0.4 RU to 1.0 RU for *Gramella forsetii*), but dropped at 85 °C (0.2 RU), mainly due to cell lysis (Additional file 11: Figure S11). This was consistent for all four tested strains which were ethanol-fixed, except for *Maribacter forsetii* for which cell deterioration already set in at 65 °C. The final optimized protocol consisted of 65 °C denaturation for 30 min, hybridization for 2 h in buffer B and 120 min of amplification. With all modifications made to the original HCR-FISH protocol from Yamaguchi et al. [21], the signal increased more than 5-fold for the same batch of 1% formaldehyde fixed *Gramella forsetii* from 0.2 to 1.0 RU, equivalent to 9 to 52% of the CARD-FISH signal (Fig. 2). For ethanol fixed *Gramella forsetii* cells, signals increased 4-fold from 0.2 to 0.8 RU, equivalent to 70% of the CARD-FISH signal of the same batch of cells.

### Validation of optimized protocol on isolates

**Fig. 3:**
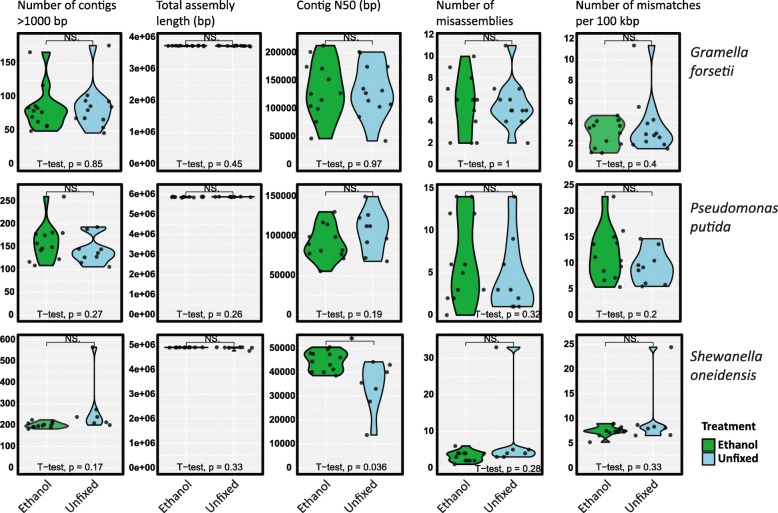
Genome quality estimation by QUAST of *Gramella forsetii*, *Pseudomonas putida* and *Shewanella oneidensis* after MDA of 500 sorted cells, either fixed with ethanol (green) or unfixed (blue). Significance thresholds (*p* values) of pairwise *t* tests are * < 0.05, ** < 0.01, *** < 0.001)

This optimized HCR-FISH protocol was applied to a set of isolates with varying GC percentages to mimic the bacterial diversity in environmental samples. The used isolates were *Gramella forsetii* (36.6% GC), *Shewanella oneidensis* (46.0% GC) and *Pseudomonas putida* (62.2% GC). The amplified DNA from 500 sorted cells, either fixed with ethanol or unfixed, were sequenced to assess genome recovery. Comparison of contig assemblies from ethanol and unfixed cells did not show significant differences except for a higher N50 for ethanol fixed *Shewanella oneidensis* (Fig. 3). Near complete genomes were recovered from the assemblies. The total assembly lengths of *Gramella forsetii* (3.8 Mbp genome size) were 3.8 Mbp (ethanol fixed) and 3.7 Mbp (unfixed), for *Shewanella oneidensis* (5.0 Mbp genome size) 4.9 Mbp from both treatments and for *Pseudomonas putida* (6.1 Mbp genome size), the assembly sizes were 5.9 Mbp from both treatments. There were no differences in read coverages between unfixed and ethanol fixed samples (Additional file 12: Figure S12).

### Proof of principle: mini-metagenomics of the uncultivated clade Vis6

We have chosen the flavobacterial clade Vis6 as a target to test our pipeline on an environmental sample. Vis6 was recurrently found in marine plankton samples from the North Sea during spring diatom blooms [24] and is without cultured representative to date. Vis6 was tracked by a nested probe approach, using a Vis6-specific HCR-FISH probe and a general *Bacteroidetes*-specific probe, which targets the majority of marine *Bacteroidetes* including Vis6.

**Fig. 4:**
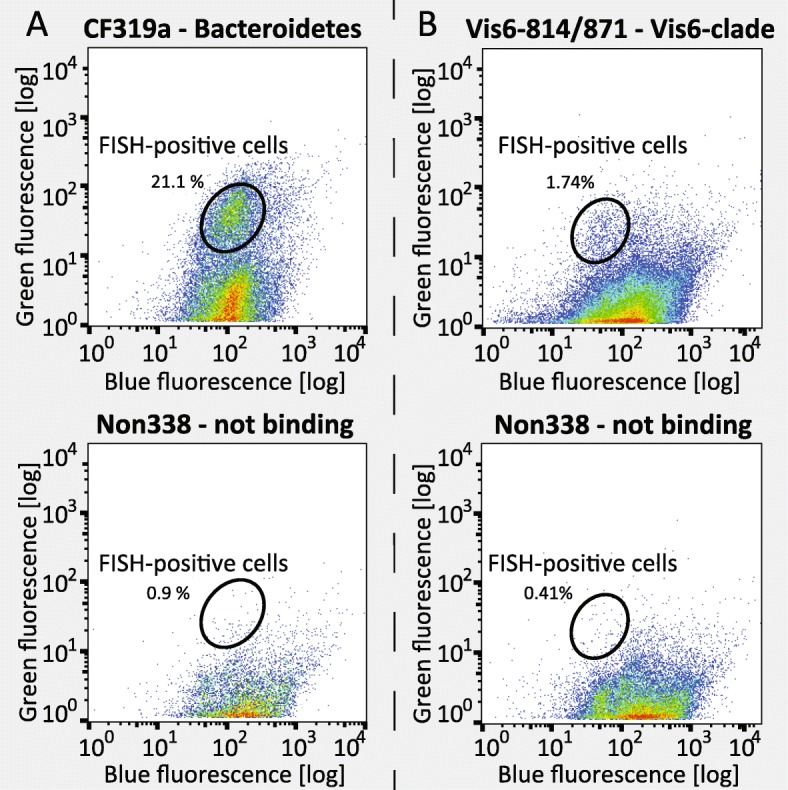
Sort criteria (gates) for flow cytometric sorting of ethanol fixed samples. A top: CF319a probe (*Bacteroidetes*); A bottom: Non338 probe (not binding, negative control); B top: Vis6-814 and Vis6-871 probe mix (Vis6-clade), B bottom: non338 probe. The blue fluorescence (355 nm laser, 460/50 nm detector) shows all bacteria stained with DAPI, the green fluorescence (488 nm laser, 530/40 nm detector) reveals probe-conferred signals. The percentage indicates the number of total events (500,000) detected within the sort gate. Note that in top panels a clearly higher percentage of FISH-positive signals for *Bacteroidetes* and Vis6 are visible above the background compared to bottom panels showing the control with the Non338 probe

CARD-FISH counts by microscopy on filtered samples showed a relative abundance of 29% for *Bacteroidetes* (targeted with probe CF319a) and of 4% for the Vis6 clade within the *Bacteroidetes* (targeted with the probe mix of Vis6-814 and Vis6-871, the helpers Vis6-814_h1 and Vis6-814_h2, and the competitor Vis6-814_c) in a seawater sample from September 20, 2017. Flow cytometric analysis revealed a distinct population of CF319a-positive cells with bright green fluorescence after HCR-FISH. This probe positive population was not detected by flow cytometry in the corresponding Non338-control that was hybridized with a non-binding probe (Fig. 4 and Additional file 13: Figure S13). The sample hybridized with probe mix Vis6-814/871 showed a population with fluorescence signals clearly above background in the flow cytometric dot plot. Five hundred cells were sorted of Vis6 cells using a combination of FISH- and DAPI-fluorescence sort criteria (“gates” in Fig. 4). A microscopic check of the sorted cells revealed purity above 93% based on the fraction of HCR-FISH stained cells. Sorted cells were subsequently subjected to the pipeline performing MDA amplification and genome sequencing. A whole community shotgun metagenome from the same water sample served as reference for the sorted mini-metagenomes.

From both ethanol-fixed and unfixed samples, cells were sorted based on their HCR-FISH-signal from CF319a-probe (targeting approximately 50% of all *Bacteroidetes*) and Vis6-814/871 probe (Vis6 clade). The results from the three sample types are summarized in Table 1. Sequences from the sorted cells and from the whole community shotgun metagenome were assembled and binned. Those bins are referred to as MAGs of high quality (> 90% completeness, < 5% contamination, ≥ 18 tRNAs), medium quality (> 50% completeness, < 10% contamination), or low quality (< 50% completeness, < 10% contamination), according to Bowers et al. [28]. From the shotgun metagenome, 4 MAGs of high quality and 11 MAGs of medium quality were retrieved, which were affiliated with several families (Additional file 14: Table S1). Of these, 1 MAG, Bin28, was classified as Vis6 with an estimated completeness of 86.4%, contamination of 2.8%, 17 tRNAs and a bin size of 1.82 Mbp. From the *Bacteroidetes*-specific sorts with probe CF319a, 1 medium quality *Bacteroidetes* MAG (51% completeness) was retrieved. It was classified as a member of the *Flavobacteriaceae*. One medium quality MAG (62% completeness), classified as *Bradyrhizobium* sp., was obtained from the negative control (sheath fluid).

**Table 1:** Comparison of high and medium quality MAGs from whole community shotgun metagenome to the mini-metagenomes from the *Bacteroidetes* (CF319a) and Vis6 (subset of *Bacteroidetes*) enrichments by HCR-FISH&FACS

	Whole community	*Bacteroidetes* (CF319a sorts)	Vis6 (Vis6-814&871 sorts)
MDA	No	Yes	Yes
Replicates	1	10	10
Sequenced reads (per replicate)	115 M	15 M	12 M
Number of MAGs (total)	15	1	7
Number of Vis6-MAGs included	1	0	7
16S rRNA in Vis6 MAG	No	No	Yes

**Fig. 5:**
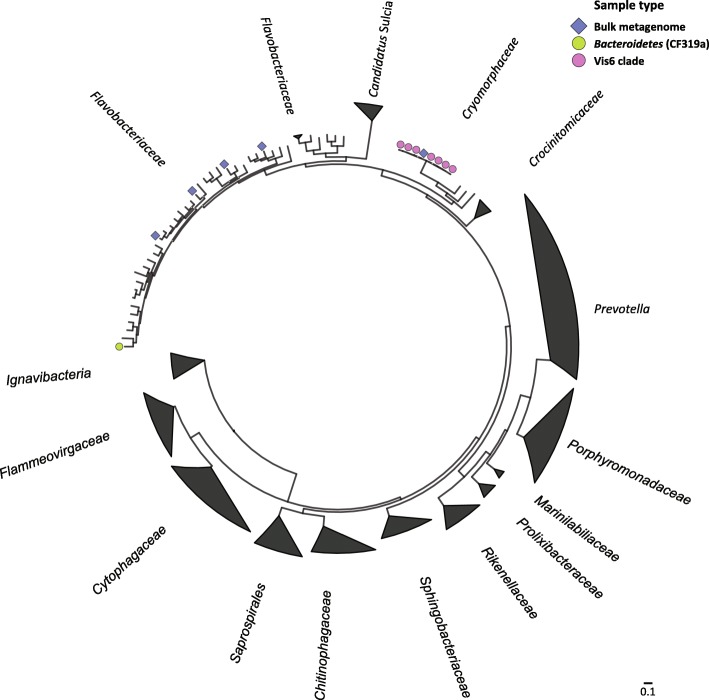
*Bacteroidetes* genome tree inferred by maximum likelihood phylogeny containing MAGs (> 50% completeness, < 10% contamination) from the bulk metagenome (blue diamonds), the CF319a sorts (green circle) and the Vis6 sorts (pink circles). The tree is based on 56 marker genes and reference sequences were pre-clustered based on the RNA polymerase

From the Vis6 specific sorts (10 replicates), MetaBAT binned four medium quality MAGs (65–82% completeness) which were classified as Vis6 (Additional file 14: Table S1). Anvi’o was used as a second, manually curated binning approach to bin the sorted Vis6 assemblies. Seven MAGs of medium quality (61–88% completeness, 0.8–3.2% contamination, 15–18 tRNAs) were retrieved which were all classified as Vis6 and shared ≥ 99% ANI (average nucleotide identity) with each other (Additional file 15: Table S2). The Anvi’o binned Vis6 MAGs had on average a higher completeness compared to the MetaBAT. MAGs from the same assembly (Additional file 16: Table S3) were used for further analysis and comparison. We retrieved more medium quality MAGs from ethanol fixed (5) than from unfixed samples (2). The Anvi’o binned MAGs and the Vis6 MAG from the shotgun metagenome were highly similar (≥ 99% ANI), suggesting each MAG represents an assembly variation of the same Vis6 population. The closest relative on genome level comparison was *Owenweeksia hongkongensis* with 49.6% average amino acid identity (AAI) (Fig. 5). Three MAGs from the Vis6 sorts, from the same population, of low completeness (< 5% contamination, 40–50% completeness) that were classified as Vis6 were included in genome annotation analyses, resulting in 10 Vis6 MAGs from the 10 Vis6 sorts in total. An overview of all MAGs classified as Vis6 is given in Additional file 17: Table S4.

Fifty complete or fragmented 16S rRNA gene sequences were retrieved from the 10 sorted Vis6 assemblies (Additional file 18: Table S5). Nine 16S rRNA gene sequences were binned into Vis6 MAGs, of which 6 sequences affiliated to the Vis6 cluster C (Additional file 19: Figure S14). Three of the sequences were found closely affiliated to *Flavobacterium ponti*. Sequences from the sorted Vis6 assemblies that were not binned in the Vis6 MAGs were mainly found closely related to *Candidatus* Brownia rhizoecola, *Flavobacterium ponti*, or *Schleiferia thermophila*. Within the whole community shotgun metagenome assembly 595 16S rRNA gene sequences were found (Additional file 20: Table S6). No 16S rRNA gene sequence was binned to the Vis6 MAG, but one complete and two partial Vis6 16S rRNA gene sequences were found within the whole assembly. The closest relative of the Vis6 clade within the 16S rRNA gene reference tree was the genus *Phaeocystidibacter* with 90% identity to the Vis6 cluster (Additional file 19: Figure S14).

39.3% of the reads from unfixed Vis6 sorts and 54.6% of reads from ethanol fixed Vis6 sorts were mapping to the Vis6 MAGs. Of the shotgun metagenome reads, 0.57% mapped back to the Vis6 bin (Bin28).

**Fig. 6:**
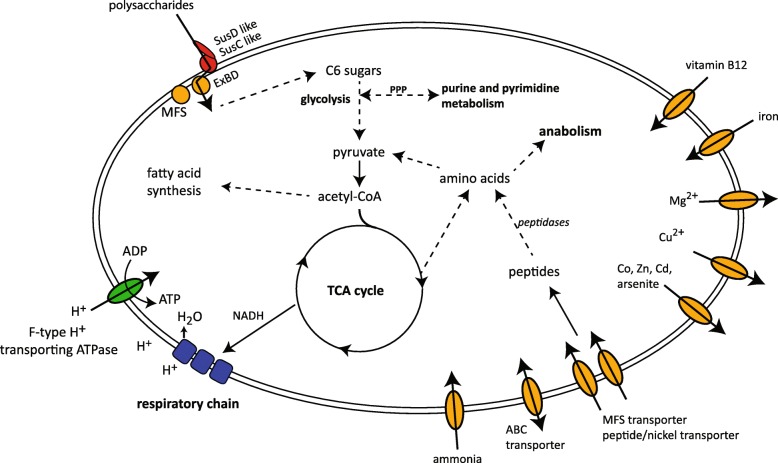
Reconstructed metabolism of Vis6 based on gene annotations of the sorted Vis6 MAGs. Genes for TCA cycle, respiratory chain, fatty acid metabolism, peptide degradation, polysaccharide uptake, and diverse transporters were annotated. MFS = major facilitator superfamily, Sus = starch utilization system, PPP = pentose phosphate pathway

From the Vis6-targeted sorts, MAGs retrieved from Anvi’o binning were used for gene annotation, in addition to three low quality Vis6 MAGs with 43–49% completeness. Genes required for core metabolism like glycolysis, citrate cycle, the non-oxidative part of pentose phosphate pathway, and fatty acid metabolism were present (Fig. 6, Additional file 21: Table S7). ABC-transporter and transporters for trace metals (Co, Zn, Cd, Mn, Fe, Ni, Mg) were found as well as phospholipid and vitamin B12 transporter (Additional file 22: Table S8). Interestingly, genes coding for bacteriorhodopsin were annotated in 4 out of 10 MAGs as well as in the metagenome MAG. Thirty-five peptidases per Mbp and 18 carbohydrate-active enzymes (CAZymes: GH, PL, CE) per Mbp were detected in the MAGs as well as a range of plasmid proteins and integrases. There were no complete sets of phage genes recovered in the Vis6 MAGs, suggesting the absence of prophages. Several integrases and transposases were found, but the MAGs were lacking genes for capsids, virus polymerases and tail fibers located in proximity to each other.

The orthofinder analysis on the Vis6 MAGs from the sorted enrichments and from the shotgun metagenome defined 5588 ortholog groups (Additional file 23: Table S9). If the ortholog group was present in at least 5 of the 10 replicates, it was defined as present in the sorted MAGs. Three hundred eighty-two ortholog groups were present in the sorted Vis6 MAGs, which were absent in the shotgun metagenome Vis6 MAG. 113 were present in the shotgun metagenome Vis6 MAG, but not in the sorted Vis6 MAGs.

## Discussion

We have successfully developed a pipeline for the targeted enrichment of uncultivated bacterial clades based on HCR-FISH and FACS, enabling subsequent whole genome sequencing and the retrieval of MAGs for genomic annotation and characterization. This pipeline was used to gain access to the yet uncultured phytoplankton bloom associated flavobacterial clade Vis6. The key steps in optimization were the cell fixation and the HCR-FISH protocol adaptations.

Samples are often taken in remote places with poor infrastructure which prevents immediate processing and makes cell fixation necessary. Since fixation stabilizes cell integrity and also permeabilizes the cell walls, less cell loss and better signal strengths could be expected after FISH for fixed cells. Therefore, we sought for a fixation protocol compatible with HCR-FISH and downstream DNA sequencing. Formaldehyde is the cell fixation of choice for microscopic analyses of FISH stained bacteria or tissues as it preserves the cell morphology well by cross-linking proteins (reviewed by [29]). Even after harsh permeabilization treatments as involved in CARD-FISH or heating of formaldehyde-fixed cells up to 85 °C as done in our study, no disruption of cells was visible. On the contrary, the signal intensity of FISH was often increased. However, we could confirm the findings of previous studies which showed that formaldehyde is not compatible with whole genome amplification and sequencing [23]. We achieved very high FISH signals and cells were easy to sort based on these fluorescence signals, but we never obtained any MDA products in repeated experiments. Aldehydes degrade DNA and form crosslinks [30, 31] which likely renders the DNA inaccessible for enzymes such as the strand displacement polymerase used during MDA.

Cell fixation with ethanol proved to be a very good alternative to formaldehyde, showing HCR-FISH signal intensities comparable to formaldehyde and the best genome assemblies in our study. The genome assembly metrics from 100 and 500 ethanol fixed cells were comparable to unfixed control samples, which is contradictory to findings from single cell genomics [23]. We also retrieved more medium quality MAGs from ethanol fixed Vis6-targeted sorts (5 MAGs with 66–88% completeness) than from unfixed Vis6-targeted sorts (2 MAGs with 61–66% completeness). This supports the results of other studies in which ethanol was a successful fixative for example for preservation of tissue morphology and RNA [32] or for PCR and sequencing from ethanol fixed tissues [33, 34].

We found that using 500 enriched cells from the same target taxa resulted in superior genome quality compared to 100 cells. Using 500 cells proved thus to be a good compromise between metagenomics and single cell genomics. Whole genome amplification methods such as MDA are widely used to amplify the small amounts of DNA from single cells for sequencing, but MDA is sensitive to contamination, especially with little input material [5, 35, 36]. This problem can be overcome by combining sequence data from multiple single cells of the same species [7] or increasing the MDA input [15]. Yu et al. [37] showed that MDA with samples of a reduced diversity increased the genome coverage of the assembled genomes. By sorting 500 cells of a specific population, the MDA input was increased and at the same time the diversity decreased. To avoid genome amplification bias completely, samples would need to be directly sequenced. The Nextera XT library preparation kit allows sequencing from low DNA input [1, 2]. Sorting at least a million cells and omitting the MDA step could be an approach to achieve highly complete MAGs without the otherwise introduced MDA bias. However, this may not be practical for most cases due to limitations in the amount of sample and long flow sorting periods in the range of many hours.

The HCR-FISH protocol modifications resulted in signal strengths, which were comparable to CARD-FISH signals. The largest signal increase was achieved by adding a mild denaturation step (65 °C) to the protocol, a step that was adopted from the geneFISH protocol [38]. The rationale behind the introduction of this step was that initiator probes are typically 49-mers and often form secondary structures, which need to be denatured to ensure an effective binding to the rRNA. We did not detect negative effects on cell morphology and on the quality of DNA when incubating samples for 30 min at 65 °C. Also, when comparing unfixed samples after HCR-FISH to unfixed, unhybridized samples we did not see any effect on genome assembly results. Thus, unlike Clingenpeel et al. [23] observed for CARD-FISH, there was no evidence for a potential DNA damage caused by HCR-FISH.

Optimal cell fixation is dependent on the target organism. Consequently, each organism needs a few adaptions to the HCR-FISH protocol, like the optimal denaturation temperature (e.g., *Maribacter forsetii* was showing decreased signals at 65 °C denaturation compared to 55 °C) or whether cell permeabilization is needed (e.g., the Gram-positive *Micrococcus* sp.). However, we are confident that the developed protocol is suitable to target diverse microorganisms from a wide range of environments. Our results also illustrated that Lugol’s solution showed sufficiently high HCR-FISH signals for detection of three out of four tested isolates and the genome assembly quality was good, though inferior to ethanol fixed cells. Still, Lugol’s solution if often used for fixation of phytoplankton samples [39] or ciliate samples [40–42] and the developed pipeline could probably be adopted for such samples as well.

The sorting of HCR-FISH targeted cells was achieved with high purity. The microscopic inspection of sorted cells indicated a sorting purity well above 90%, based on FISH positive signals. Similarly, 23 out of 25 MAGs from *Bacteroidetes* sorts that could be classified by checkM (taking also low quality MAGs with < 50% completeness into account) were classified as *Bacteroidetes*, indicating a high sorting purity on the level of sequencing as well. With 0.57% of the metagenome reads mapping to the Vis6 bin and 47% of the Vis6-sort reads mapping to the Vis6 bins from the sorted fraction, the enrichment approximates 80-fold. These enrichment values are estimates due to the potential amplification bias of MDA, but a high enrichment of the targeted group is apparent. This sorting approach thus allows for replicates which are usually not done for sequencing intensive metagenomics.

Such replicates could also enable the detection of lower frequency genes that are collapsed to a consensus genome in whole community shotgun MAGs. We found three times more ortholog groups present in the sorted Vis6 MAGs, that were absent in the Vis6 MAG from bulk shotgun sequencing, than ortholog groups, that were present in the Vis6 MAG from bulk shotgun sequencing, but absent in the sorted Vis6 MAGs. Still, a high microdiversity within a sorted population which cannot be distinguished based on the limited taxonomic resolution of the 16S rRNA will most likely not be uncovered by our pipeline. Repeated sorting of single cells based on the HCR-FISH signal of specific probes could potentially cover such species and dissect strain heterogeneity. The drawback would be that SAGs suffer from reduced genome coverage, but adopting our pipeline to generate several SAGs from the same species by repeated sorting would enable us to study species and strain heterogeneity.

In 6 out of 10 MAGs from sorted Vis6 cells, the respective 16S rRNA sequence was binned, which was not observed in the single MAG from the whole community shotgun metagenome. However, two of the sorted Vis6 bins had an additional 16S rRNA gene belonging to *Flavobacterium ponti*, suggesting that 16S rRNA binning was inaccurate. It is unlikely that *F. ponti* was hybridized by the Vis6 probes because Vis6-871 had 4 and Vis6-814 had 5 mismatches with the 16S rRNA gene sequence of *F. ponti*, which basically excludes binding of the probes under the used hybridization conditions. We assume that the 16S rRNA gene was falsely binned due to its high conservancy. Nevertheless, a quite high reliability for the 16S rRNA gene identity to the MAGs is given by the 16S rRNA sequence being binned in the replicates and by the 16S rRNA sequence targeted probe that was used for sorting. Microscopic verification that the probe had hybridized to the sorted cells provides further certainty.

From both the whole community metagenome and the Vis6-targeted sorts, MAGs with low contamination (< 5%) and completeness values > 80% were retrieved. This shows that the developed pipeline is producing MAGs of sufficient quality for the description of uncultivated microorganisms as *Candidatus* species, comparable to, e.g., Francis et al. [43]. The genome size (estimated from total contig length and completeness estimation) was in the range of 2.1–2.4 Mbp for all of the Vis6 MAGs. The closest isolated relative based on the whole genome analyses was *Owenweeksia hongkongensis* to which the sorted MAGs had an amino acid identity (AAI) of 49.6%, indicating that Vis6 is a novel genus within the *Cryomorphaceae* [44].

With the annotation of the Vis6 MAGs obtained from our analyses, we were able to reconstruct main metabolic pathways, hypothesizing an aerobic lifestyle. This lifestyle is mainly based on the consumption of polymeric substrates like polysaccharides and peptides, indicated by the annotation of peptidases and CAZymes. Closely related flavobacterial species are known to be specialized on these polymers as well [43, 45, 46]. As the Vis6 clade was detected during phytoplankton blooms in spring times [24], a consumption of substrates secreted by living and released by decaying phytoplankton seems likely [47]. The higher percentage of peptidases compared to CAZymes and the rather small genome of Vis6 is similar to other phytoplankton bloom responders [46, 47]. Future CAZyme analyses will provide more detailed information about the type of polysaccharides that Vis6 can degrade, like it has been analyzed for example for *Formosa* species [48, 49]. Besides, the annotations of ABC-type transporters point to a utilization of low molecular weight substrates. Such a broad metabolic repertoire could indicate a flexible lifestyle on diverse substrates. The detection of bacteriorhodopsins in some of the MAGs indicate the ability to sustain life under substrate depletion [50]. Most likely our organism is a free-living bacterium since it was sorted from the 3-μm-prefiltered size fraction of the picoplankton and was never found attached to particles in unfractionated samples by FISH.

## Conclusions

The link between metabolic functions and taxonomic identity is not always given by metagenomic sequencing, but important for understanding ecosystem processes. Single cell genomics can provide this link, but the assembled genomes are usually incomplete. We developed a targeted pipeline, combining HCR-FISH and FACS, which offers the opportunity to enrich an organism of interest from the environment and sequence a mini-metagenome of reduced taxonomic diversity. The resulting metagenome assembled genomes are of higher completeness than SAGs and offer the analysis of replicates. The pipeline could be especially useful in high diversity ecosystems where assembly of low abundance organisms is hampered using standard metagenomics. Other environments like sediment or soil samples might be amenable with our protocol as well given a thorough separation of cells from particles can be achieved. This can be tested in future studies as well as the targeted sorting based on functional genetic markers using geneFISH [38].

## Materials and methods

### Bacterial cultures

Cultures for the cell fixation experiment were grown on a shaker to an OD_600_ of ~ 0.1 under different growth conditions (Additional file 24: Table S10) before cells were fixed. The different fixatives and incubation times for cell fixation are given in Table 2. Formaldehyde (4%, 1%, 0.25%, 0.1%; vol/vol), glyoxal (4%, 4% + 5% ethanol) or Lugol’s solution (Sigma Aldrich, L6146-1L; 2%, 2% + 0.07 M sodium-thiosulfate) was added to culture aliquots and cells were harvested by centrifugation after the given incubation time. The resulting cell pellets were washed once in 1× PBS (phosphate-buffered saline) and finally resuspended in 1× PBS and stored at 4 °C. Ethanol fixation was done by first harvesting the cells in a pellet, then resuspension in 70% ethanol in 1x PBS, incubation at RT for 1 h before storage at 4 °C. Sodium-thiosulfate for destaining of Lugol’s solution was added as the last step prior to storage. The fixed cells were filtered within a few days on 0.2 μm polycarbonate filters (Isopore™ Membrane filters, Merck Millipore, Ltd.) using a vacuum pump (200–300 mbar), air dried and stored at − 20°C.

**Table 2:** Fixatives and fixation times used on pure cultures

	Fixative	Concentration	Temperature	Time
1	Formaldehyde	4%	Room temperature	1 h
2	Formaldehyde	1%	Room temperature	1 h
3	Formaldehyde	0.25%	Ice	10 min
4	Formaldehyde	0.1%	Ice	10 min
5	Ethanol	70%	Room temperature	1 h
6	Lugol’s solution	2%	Room temperature	1 h
7	Lugol’s and sodium thiosulfate	2% and 0.07 M	Room temperature	1 h
8	Glyoxal	4%	Room temperature	1 h
9	Glyoxal and ethanol	4% and 5%	Room temperature	1 h
10	Control	–	–	–

Cultures for HCR-FISH optimization were grown according to Additional file 24: Table S10 and ethanol fixed as described above. Additional aliquots of *Gramella forsetii* were fixed with 1% formaldehyde (10 h at 4 °C). Cells were filtered on 0.2 μm polycarbonate filters (Isopore™ Membrane filters, Merck Millipore, Ltd.), air dried and stored at − 20 °C.

### Environmental samples

Water samples from Helgoland (station “Kabeltonne”, 54° 11.34′ N, 7° 54.04′ E) were collected on September 20, 2017 by the research vessel Aade (https://www.awi.de/en/expedition/ships/more-ships.html). Water was filtered through 10 μm and 3 μm Isopore™ Membrane filters (Merck Millipore Ltd.) to remove larger particles before cells were collected on 0.2 μm polycarbonate filters (Isopore™ membrane filters, Merck Millipore Ltd.). Environmental samples were used either unfixed or ethanol fixed. For ethanol fixation the filters were incubated in 96% ethanol for 15 min directly after filtration. All filters were air dried and subsequently stored at − 20 °C.

### FISH methods

FISH with 4× fluorescently labeled oligonucleotide probes was performed as described in Fuchs et al. [51] and CARD-FISH control experiments were done according to Pernthaler et al. [18]. All probes used in the experiments are listed in Additional file 25: Table S11.

The HCR-FISH protocol was modified after Yamaguchi et al. [21] and performed on filters. The gram-positive *Micrococcus* sp. KT16 was permeabilized prior to hybridization by incubation in a buffer containing 0.7 mg ml^-1^ lysozyme, 0.5 M EDTA, and 1 M Tris-HCl [pH 7.5] at 37 °C for 8 min. All other cells were used without enzymatic permeabilization. Filter pieces were covered with hybridization buffer A (1 μM probe, 20 mM Tris-HCl [pH 7.5], 0.9 M NaCl, 0.01% SDS, 10% dextran sulfate, 1% blocking reagent, 35% formamide) and placed in a humidified chamber (containing a 35% formamide water mixture) at 46 °C for 2–3 h. To remove excess probe, the filters were washed in washing buffer (20 mM Tris-HCl [pH 7.5], 0.01% SDS, 0.08 M NaCl) at 48 °C for 20–30 min. The NaCl concentration in the washing buffer varies with the formamide concentration in the hybridization buffer [52]. During the washing step, the fluorescently labeled hairpin oligos H1 and H2 were prepared: H1 and H2 (4× labeled with Atto488, Biomers, Ulm, Germany) were separately diluted (5 μM) in amplification buffer (50 mM Na_2_HPO_4_, 0.9 M NaCl, 0.01% SDS, 1% blocking reagent, 10% dextran sulfate), heated in a thermocycler to 95 °C for 1.5 min and cooled to 25 °C for 1 min. H1 and H2 were mixed shortly before usage. After washing, the filter pieces were covered with the mixed H1/H2 solution and placed in a humid chamber at 37 °C for up to 2 h. Following this amplification step, the filter pieces were washed twice in ice-cold 1× PBS for 5 min and subsequently 30 s in ice-cold ultrapure water. Filters were then air dried and processed for microscopy or cell sorting or stored at 4 °C if used the next day.

The optimization parameters tested on pure cultures included the addition of a denaturation step before hybridization where filters were placed in the hybridization chamber, covered with hybridization buffer and probes, and incubated at temperatures ranging from 45 °C to 85 °C for 30 min prior to hybridization at 46 °C. In addition, hybridization buffer B was tested as an alternative to the hybridization buffer A as described by Barrero-Canosa et al. [38] (5×SSC (750 mM NaCl, 0.075 mM sodium citrate), 20% dextran sulfate, 0.1% SDS, 20 mM EDTA, 0.25 mg ml^−1^ sheared salmon sperm DNA, 0.25 mg ml^−1^ yeast RNA and 1% blocking reagent for nucleic acids (Roche, Basel, Switzerland)). Finally, different chain reaction signal amplification times of 15–120 min were tested.

### Microscopy

The impact of the tested HCR-FISH protocol parameters on per cell fluorescence was determined by microscopy. Pure cultures, fixed with 70% ethanol or 1% formaldehyde were used. All samples were counterstained with 4′,6-diamidino-2-phenylindole (DAPI) before observation under an automated epifluorescence microscope (Zeiss Axioplan2 imaging, Carl Zeiss, Oberkochen, Germany) equipped with a monochrome camera (AxioCam MRm, Carl Zeiss Microimaging GmbH, Göttingen, Germany). More than 1000 DAPI stained cells were counted to calculate the relative abundances of targeted groups and all the experiments were performed in duplicates. For relative brightness evaluation the Inspeck Green Microscope Image Intensity Calibration Kit (Molecular Probes, Eugene, Oregon, USA) was used following manufacturer’s instructions. The signal intensity of the FISH positive cells was calculated using the digital image analysis software program ACMEtool2 [53] on more than 1000 single cells for each experiment. Signal intensity was expressed as relative unit (RU).

### Flow cytometric analysis

After HCR-FISH on filters, the cells were vortexed for 15 min at 4 °C in 1.5 ml buffer [14] to detach the cells from the filter in solution for flow cytometry. Environmental samples were incubated in the buffer for 30 min at 37 °C prior to vortexing at RT [14]. The filter pieces were removed and cells in suspension were stored up to 1 day at 4 °C in dark until cell sorting.

Samples for flow cytometric analysis were counterstained with DAPI solution, 1–2 μg ml^−1^ final concentration. The samples were recorded and sorted with a BD Influx™ system (BD Biosciences, San Jose, USA) with the BD FACS™ Sortware v1.2 with a 86 μm nozzle, 0.15% NaCl solution as sheath fluid and equipped with a 488 nm (200 mW) and 355 nm (100 mW) laser (Coherent, Dieburg, Germany). The analysis was done with FlowJo® v10 software (FlowJo, LLC). Multifluorescent beads (1 μm, FluoresbriteR, Polyscience Inc.) were used for optical alignment. The trigger was set to green fluorescence for pure cultures and to forward scatter for environmental samples. Pure cultures were sorted by selecting the population containing the cells in the green fluorescence FISH (530/40 band-pass filter) vs. blue fluorescence DAPI (450/60 band-pass filter) plots. For environmental samples, a parent gate in the FSC vs. green fluorescence plot and a sub-gate in the green fluorescence vs. blue fluorescence plot was used for sorting (Additional file 26: Figure S15). To ensure that the flow cytometer was running stable, we compared bead signals at the start and end of an experiment day. The signals recorded at the same day are thus comparable. The signals recorded at different days are not absolutely comparable, because they are not calibrated on beads. All samples from the same organism were recorded on the same day. So were the environmental samples. For sheath control, no sample was running and machine noise signals were used to sort a specified number of events. Cells were sorted in 384 well plates (LightCycler®, Roche Molecular Systems Inc.).

Samples from the first fixation benchmarking of isolates and the environmental samples were sorted at the MPI (Bremen) and the verification experiment was done at the JGI (Walnut Creek). For the latter, the preparations and sorting parameters vary therefore to those described above. Cells were vortexed for 5 min at room temperature in buffer [14] to detach the cells from the filter and immediately processed. DNA-staining was done with Syto59 (0.5 μm) and sorted with a BD Influx™ system with a 70 μm nozzle and 1x PBS as sheath fluid. Sorting was based on gating the Syto59 population (670/30 detector, 642 nm laser) and the FISH signal (530/40 detector, 488 nm laser).

### Amplification, sequencing, and assembly

All sorted cells were amplified with multiple displacement amplification (MDA) with Single cell REPLI-g (QIAGEN, Venlo, Netherlands) for 6 h. Sequencing libraries were created with Nextera XT v2 with 9 rounds of PCR and sequenced on the Illumina NextSeq-HO (2 × 150 bp read length). Reads were assembled with SPAdes assembler 3.11.1 [54] and analyzed with Quast v5.0.2 [55].

For the whole community shotgun metagenome, DNA was extracted from an unfixed filter from September 20, 2017 using the PowerSoil® DNA Isolation Kit (Mo Bio Laboratories, Inc., Carlsbad, USA) and the DNA was sequenced with Illumina NextSeq-MO (2 × 150 bp length) sequencing method.

The isolate *Micrococcus* sp. KT16 was genome sequenced under the GOLD [56] analysis ID Ga0256418 with the sequencing methods Illumina HiSeq 2500-1TB and PacBio RS II.

### Contig binning

Initial binning of assembled contigs was done with MetaBAT2 [57]. The FISH positive samples sorted by the Vis6 probes (10 samples) were binned manually with Anvi’o [58] based on sequence identity and differential coverage information that was retrieved by mapping the reads to the respective assemblies using BBMap v35.14 (http://bbtools.jgi.doe.gov), using fast mode and setting minid and idfilter to 0.97. CheckM provided an approximate taxonomic classification and genome quality estimation [59].

### Phylogenomic analysis

A reference genome tree was constructed based on reference genomes of the phylum *Bacteroidetes* with RNA polymerase as marker gene with 90% clustering to reduce the dataset. The RNA polymerase sequences were collected with hmmsearch v3.1b2 [60] and aligned with MAFFT [61] using the mafft-linsi option. Sites with 90% gaps were trimmed with trimAl 1.4 [62]. Genomes not containing all three subunits of the RNA polymerase were excluded. The genome tree was calculated with maximum likelihood phylogeny with IQ-tree [63], using the WAG substitution model and 1000 bootstraps and visualized in ggtree [64].

### Read recruitment

For read recruitment, error corrected reads from the sorted Vis6 samples were mapped back to the Vis6 bins with BBMap v35.14 as described above. The whole community shotgun reads were mapped back to the metagenome Vis6 bin.

### Orthofinder

Orthofinder [65] was run on all Vis6 MAGs from Vis6 sorts and whole shotgun metagenome. These genomes fell within 95% ANI of one another and were passed as input to Orthofinder 2.2.7 with the run line: OrthoFinder-2.2.7/orthofinder-f ExampleDataset-S diamond. Gene families were created and their presence and absence in the Vis6 MAGs from sorts and shotgun sequencing analyzed.

### Gene annotation

For gene annotation all bins were processed by the IMG annotation pipeline [66] and are available by the ER comparative analysis system IMG/MER [67] under the GOLD [56] Study ID Gs0130320. The KEGG predictions from IMG were used to look for metabolic pathways (www.genome.jp/kegg). Transporter and specific proteins were searched text-based. Peptidases were annotated by BLAST against the merops database [68], and carbohydrate-active enzymes (CAZymes) were annotated using the dbCAN v6 database [69]. Phaster [70] and VirSorter [71] were used to check for viral sequences and prophages within the Vis6 MAGs. The average nucleotide identity (ANI) and amino acid identity (AAI) between MAGs and references were calculated using ani.rb and aai.rb from the enveomics collection [72].

### 16S rRNA analysis

16S rRNA gene sequences were detected in the assemblies using the ssu_finder option in checkM [59] and aligned with ACT implemented on www.arb-silva.de. A reference tree was created based on the SILVA database release 128 SSU Ref (www.arb-silva.de) with sequences from Helgoland clone libraries added [73]. Analyses were done with the ARB software [74]. All sequences within the “uncultivated” cluster of the *Cryomorphaceae* that included Vis6 sequences (tested by probe match function of ARB) were selected in addition to isolate sequences from class *Flavobacteriia* as outgroup. Following the guideline of Peplies et al. [75], four different trees were calculated (neighbor joining and RaXml using termini filter, with and without 30% *Bacteroidetes* filter) and a consensus tree created. 16S rRNA sequences from the assemblies were added to the tree using the ARB parsimony (quick add) function.

## Supplementary information

**Additional file 1: Figure S1.** The development of our pipeline was done in three steps before the application on an environmental sample. (1) In the first step, four bacterial isolates (GC content is given in mol%) were treated with four different fixatives (plus unfixed control) and their signal intensity was measured by flow cytometry (1a). Glyoxal was not further analyzed due to low signal intensities (red cross). Three isolates and three of the brighest fixations (plus control) were sorted (100 and 500 cells) and forwarded to MDA (1b). The MDA products of two isolates were sequenced and their genome quality assessed (1c). The best results in total (signal intensity and genome quality) were achieved with ethanol fixation and 500 cells (green star). (2) In the second step, the HCR-FISH protocol from Yamaguchi *et al.* [21] was adapted with different denaturation temperatures, hybridization buffers and amplification times. The signal intensities were assessed after HCR-FISH via microscopy. (3) In a third step, the optimized HCR-FISH protocol was tested for validation on isolates with ethanol fixation (plus unfixed control). There were no significant differences in assembly metrics between ethanol fixation and unfixed control samples after sequencing of MDA products from 500 sorted cells. (4) The optimized HCR-FISH protocol was tested on ethanol fixed and unfixed seawater samples. *Bacteroidetes* and the flavobacterial clade Vis6 were targeted by specific HCR-FISH probes, 500 cells sorted and sequenced. For comparison a whole community shotgun metagenome was prepared.

**Additional file 2: Figure S2.** The background fluorescence in the green channel of flow cytometric measurements was set to 10 RU (blue line), based on comparisons betwen EUB-338 and Non-338 probes hybridized to 1% formaldehyde fixed *Gramella forsetii* (A, B) and *Micrococcus* sp. (C, D) samples. The Non-338 control of *Gramella forsetii* (A) was not stained with DAPI, the Non-338 control of *Micrococcus* sp. (C) was stained with DAPI. Green fluorescence was detected with a 530/40 nm filter, blue fluorescence with a 460/50 nm filter. 5000 events were recorded for *Gramella forsetii* (A, B) and 2000 for *Micrococcus* sp. (C, D).

**Additional file 3: Figure S3.** Four pure cultures (*Gramella forsetii*, *Maribacter forsetii*, *Escherichia coli*, *Micrococcus* sp.) were fixed with 10 different fixation methods (formaldehyde 4%, 1%, 0.25%, 0.1%, ethanol, Lugol’s solution with and without thiosulfate, glyoxal with and without ethanol and unfixed). HCR-FISH was done on filtered cells and signal intensity was measured after washing the cells off the filter and analyzing them in the flow cytometer. Plotted are the green fluorescence (530/40 nm) from HCR-FISH and blue fluorescence (450/60 nm) from DAPI staining. The fluorescence intensity is given in relative units on a logarithmic scale. The background fluorescence (dotted line) was defined for 10 RU. N.A. = not analyzed due to disrupted cells.

**Additional file 4: Figure S4.** Blue fluorescence intensity (DAPI signal, 450/60 nm band-pass filter) of four isolates in dependency of cell fixation, measured by flow cytometry. The median of the signal population from flow cytometric analysis is shown. NA = not analyzed due to disrupted cells.

**Additional file 5: Figure S5.** Microscopic images of differently fixed *Gramella forsetii* cells after cell sorting. Shown are overlay images of the HCR-FISH signal (green) and DAPI signal (blue). All images were taken with an epifluorescence microscope with a HC409LP (DAPI) and an ET500/LP (HCR-FISH) filter. The numbers in brackets indicate the exposure time for each image with the first number corresponding to the DAPI and the second to the HCR-FISH signals.

**Additional file 6: Figure S6.** Exemplary images of gel electrophoresis with MDA products from sorted isolates, fixed with formaldehyde, Lugol’s solution, ethanol or unfixed. The uppermost band of the used marker LambdaDNA Hind III corresponds to 23 kb. MDA products from formaldehyde fixed cells were either not detectable or in low amount.

**Additional file 7: Figure S7.** Crossing point times (CP, time of inflection point of real time amplification curve) of MDA reactions of three isolates with 100 or 500 cells input. Input samples were taken from Lugol’s fixed, ethanol fixed and unfixed cells. Additionally, unfixed cells that have not been subjected to FISH were used.

**Additional file 8: Figure S8.** Genomic quality estimation of *Gramella forsetii* and *Maribacter forsetii* sequencing products after MDA of 100 and 500 sorted cells using Quast. Shown are from left to right: the number of contigs longer than 1000 bp, the total assembly lengths, N50, number of misassemblies and number of mismatches per 100 kpb. Unhybridized samples were unfixed and were not subjected to HCR-FISH, but only sorted based on their DAPI signal. *Maribacter forsetii* unhybridized controls were taken from a cell aliquot and have not been filtered and washed off a filter like the other controls, including the *Gramella forsetii* unhybridized control. Significance thresholds (p-values) of pairwise t-tests are * < 0.05, ** < 0.01, *** < 0.001.

**Additional file 9: Figure S9.** Read coverages across the reference genome of *Gramella forsetii* and *Maribacter forsetii* cells. 100 and 500 cells were sorted from isolates, used as input for MDA and the products were sequenced. Unfixed, unhybridized cells of *Maribacter forsetii* were taken directly from the culture without being filtrated and washed off the filter, which explains the difference in coverage between the treatments that we did not see for *Gramella forsetii*.

**Additional file 10: Figure S10.** Signal intensity of formaldehyde fixed *Gramella forsetii* cells, after HCR-FISH with different treatments. CARD-FISH and the former protocol (yellow bar: in hybridization buffer A, no denaturation, 45 min amplification) were used for comparison. 30 min denaturation at 65°C and 85°C were tested in combination with hybridization buffer A and hybridization buffer B for three amplification times: 15 min, 45 min and 120 min. The signal intensities were measured via microscopy and are given in RU. The choice of hybridization buffer did not make a significant difference to signal intensity, but HCR-FISH signals were slightly higher when buffer B was used (see materials and methods for details in composition). Increasing chain reaction amplification time from 15 or 45 min to 120 min enhanced fluorescence from 0.3 (15 min) and 0.4 RU (45 min) to 1.1 RU (average values).

**Additional file 11: Figure S11.** Signal intensity of four ethanol fixed isolates after HCR-FISH with 30 min denaturation (45-85°C) or without denaturation (-) and 2 h hybridization in comparison to CARD-FISH (CARD). Signal intensities were measured via microscopy and are given in RU.

**Additional file 12: Figure S12.** Sequencing read coverages across the reference genome of *Gramella forsetii*, *Shewanella oneidensis* and *Pseudomonas putida*. 500 cells from ethanol fixed (green) and unfixed (brown) samples were used as input for MDA.

**Additional file 13: Figure S13.** Flow cytometric sort gates of unfixed samples, targeted with A: CF319a (*Bacteroidetes*) probe and Non338 (not binding) probe and B: Targeted with the probe mix Vis6-814/871 (Vis6-clade) and Non338 probe. The blue fluorescence (355 nm laser, 450/60 nm detector) shows all bacteria stained with DAPI. The percentage indicates the number of detected events within the sort gate. The amount of signals with green fluorescence (488 nm laser, 530/40 nm detector) increase with samples targeted with a probe compared to the Non338 probe.

**Additional file 14: Table S1.** Statistics of all bins created with MetaBAT from the whole community shotgun metagenome, the CF319a sorts and Vis6-814/871 sorts. Marked in bold are those bins classified as Vis6.

**Additional file 15: Table S2.** Sample description and statistics of Anvi'o binned assemblies from sorted Vis6 samples. Marker_lineage, completeness, contamination and strain heterogeneity were assessed with checkM.

**Additional file 16: Table S3.** Comparison of Anvi’o and MetaBAT binning methods on the assemblies of sorted Vis6. Completeness, contamination and strain heterogeneity were assessed with checkM; GC content and bin size were assessed with stats.sh (Bbmap). The genome size is calculated from the bin size and the completeness values. All bins classified as Vis6 are shown, including low quality ones.

**Additional file 17: Table S4.** Draft genomes of Vis6 from Vis6-targeted sorts and from bulk metagenome.

**Additional file 18: Table S5.** 16S rRNA gene sequences of sorted Vis6 assemblies, binned with Anvi'o and retrieved with ssu_finder (checkM). The closest relative in the 16S rRNA tree was analysed by adding the sequences into the Vis6 consensus tree.

**Additional file 19: Figure S14.** 16S rRNA consensus tree. The Vis6 cluster is targeted by the probes Vis6-814 and Vis6-871 and marked by the colored box. The closest cultured relative was *Phaeocystitibacter luteus* with appr. 90% sequence similarity. Nine 16S rRNA sequences, that have been binned to Vis6 MAGs from the Vis6 sorts, were placed in this tree. Six sequences were affiliated to Vis6 cluster C (marked in pink) and three sequences were affiliated to *Flavobacterium ponti* (in the outgroup). *Flavobacterium ponti* has appr. 86% 16S rRNA sequence identity to the Vis6 cluster.

**Additional file 20: Table S6.** 16S rRNA gene sequences from the whole community shotgun metagenome, retrieved with ssu_finder (checkM), with SILVA classification. Three sequences in bold are assigned to the Vis6 cluster.

**Additional file 21: Table S7.** The KEGG mapper was used to check for presence of metabolic pathways in the sorted Vis6 MAGs. For each pathway it is indicated whether it was found completely or not.

**Additional file 22: Table S8.** The annotated Vis6 MAGs from sorted samples were analysed by text-based search for transporters using the key words transport, transporter, influx, efflux, export, import, secretion, channel, SusC, SusD, TonB, ExbB, ExbD and ABC. Additionally proteins involved in the respiratory chain were searched for. Given are the numbers found in all sorted Vis6 MAGs (10) and the number per MAG.

**Additional file 23: Table S9.** Presence absence of ortholog groups (og) of the 10 Vis6 MAGs from the sorts (B-K), the Vis6 MAG from the bulk metagenome (L) and five reference strains with IMG accession number (M-Q). Column R is the sum of the sorted MAGs (B-K).

**Additional file 24: Table S10.** Bacterial strains used for benchmarking the influence of cell fixation and for HCR-FISH optimization.

**Additional file 25: Table S11.** Probes used in FISH experiments. In HCR-FISH, the probe consists of a taxon specific sequence attached to the initiator sequence. The G’s in bold of the hairpin oligonucleotides are labelled with Atto488 (Biomers).

**Additional file 26: Figure S15.** Exemplary scatter plots from flow cytometry showing the gating principle of seawater samples hybridized with HCR-FISH probe. (A) DAPI positive cells were selected in the forward scatter vs. blue fluorescence (DAPI signal) plot and (B) HCR-FISH positive cells were selected in the blue vs. green fluorescence (HCR-FISH signal) plot. Of (B) only those events that were also appearing in the gate in (A) were sorted.

**Additional file 27.** Lists of contigs of the Vis6 affiliated bins that have been binned with Anvi’o.

**Additional file 28:.** List of contigs of the Vis6 affiliated bins that have been binned with Metabat2.

## Data Availability

Sequencing data is published under the GOLD (www.gold.jgi.doe.gov) Study ID Gs0130320. The scaffolds of each metagenome that have been binned to the Vis6 bins are listed in Additional files 27 and 28.
