# Supplementary material for: A pipeline for targeted metagenomics of environmental bacteria
Source: Microbiome. 2020 Feb 15;8:21. doi: 10.1186/s40168-020-0790-7 (PMC7024552; doi:10.1186/s40168-020-0790-7)
Supplement: Supplementary file 2 — Additional file 1: Figure S1. The development of our pipeline was done in three steps before the application on an environmental sample. (1) In the first step, four bacterial isolates (GC content is given in mol%) were treated with four different fixatives (plus unfixed control) and their signal intensity was measured by flow cytometry (1a). Glyoxal was not further analyzed due to low signal intensities (red cross). Three isolates and three of the brighest fixations (plus control) were sorted (100 and 500 cells) and forwarded to MDA (1b). The MDA products of two isolates were sequenced and their genome quality assessed (1c). The best results in total (signal intensity and genome quality) were achieved with ethanol fixation and 500 cells (green star). (2) In the second step, the HCR-FISH protocol from Yamaguchi et al. [21] was adapted with different denaturation temperatures, hybridization buffers and amplification times. The signal intensities were assessed after HCR-FISH via microscopy. (3) In a third step, the optimized HCR-FISH protocol was tested for validation on isolates with ethanol fixation (plus unfixed control). There were no significant differences in assembly metrics between ethanol fixation and unfixed control samples after sequencing of MDA products from 500 sorted cells. (4) The optimized HCR-FISH protocol was tested on ethanol fixed and unfixed seawater samples. Bacteroidetes and the flavobacterial clade Vis6 were targeted by specific HCR-FISH probes, 500 cells sorted and sequenced. For comparison a whole community shotgun metagenome was prepared. [file 40168_2020_790_MOESM1_ESM.pdf]

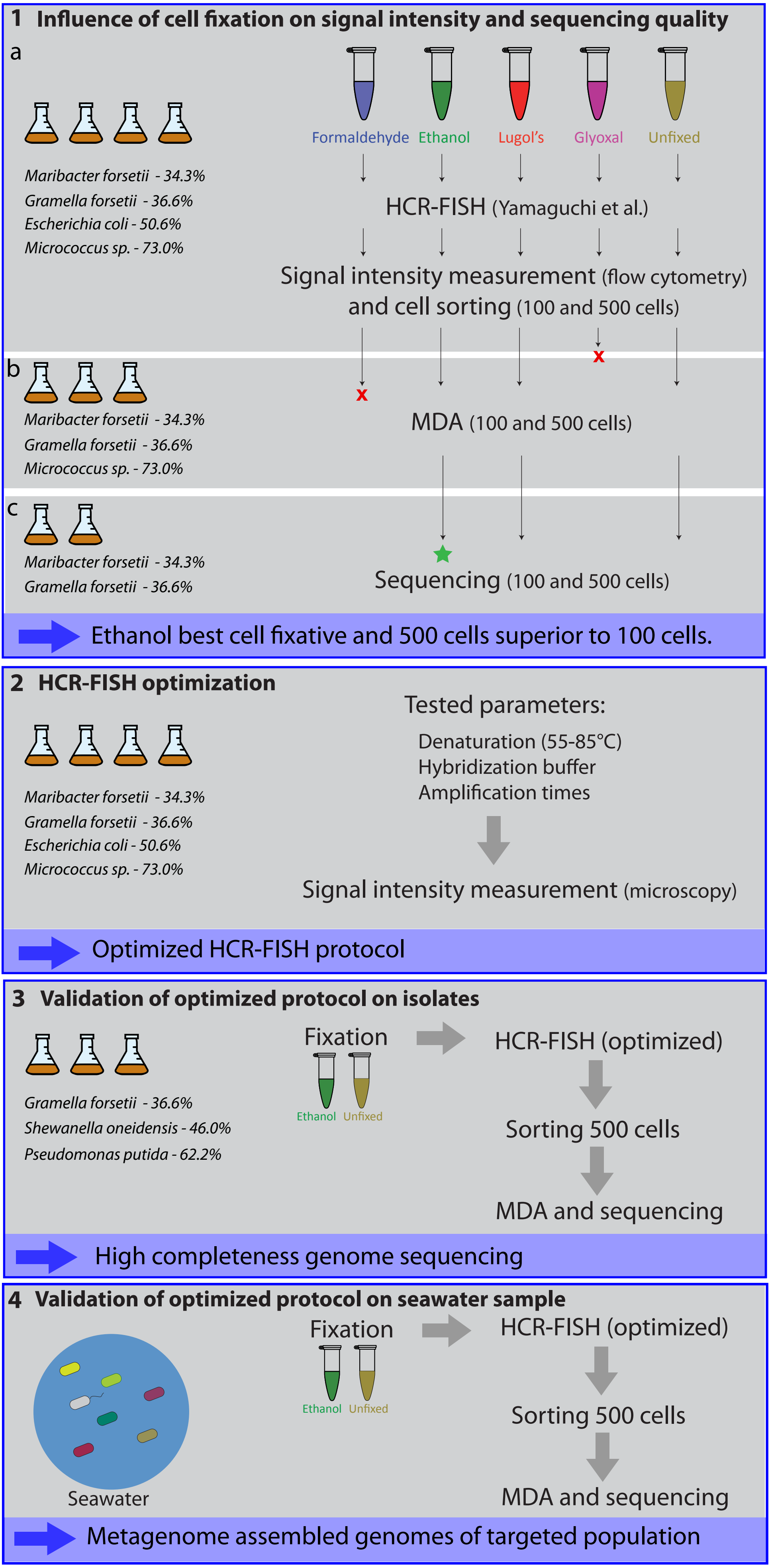
1 Influence of cell fixation on signal intensity and sequencing quality
a
Maribacter forsetii - 34.3%
Gramella forsetii - 36.6%
Escherichia coli - 50.6%
Micrococcus sp. - 73.0%
Formaldehyde
Ethanol
Lugol's
Glyoxal
Unfixed
HCR-FISH (Yamaguchi et al.)
Signal intensity measurement (flow cytometry)
and cell sorting (100 and 500 cells)
b
Maribacter forsetii - 34.3%
Gramella forsetii - 36.6%
Micrococcus sp. - 73.0%
x
x
MDA (100 and 500 cells)
c
Maribacter forsetii - 34.3%
Gramella forsetii - 36.6%
Sequencing (100 and 500 cells)
Ethanol best cell fixative and 500 cells superior to 100 cells.
2 HCR-FISH optimization
Maribacter forsetii - 34.3%
Gramella forsetii - 36.6%
Escherichia coli - 50.6%
Micrococcus sp. - 73.0%
Tested parameters:
Denaturation (55-85°C)
Hybridization buffer
Amplification times
Signal intensity measurement (microscopy)
Optimized HCR-FISH protocol
3 Validation of optimized protocol on isolates
Gramella forsetii - 36.6%
Shewanella oneidensis - 46.0%
Pseudomonas putida - 62.2%
Fixation
Ethanol
Unfixed
HCR-FISH (optimized)
Sorting 500 cells
MDA and sequencing
High completeness genome sequencing
4 Validation of optimized protocol on seawater sample
Seawater
Fixation
Ethanol
Unfixed
HCR-FISH (optimized)
Sorting 500 cells
MDA and sequencing
Metagenome assembled genomes of targeted population
