# Supplementary figures and images for: A pipeline for targeted metagenomics of environmental bacteria
Source: Microbiome. 2020 Feb 15;8:21. doi: 10.1186/s40168-020-0790-7 (PMC7024552; doi:10.1186/s40168-020-0790-7)

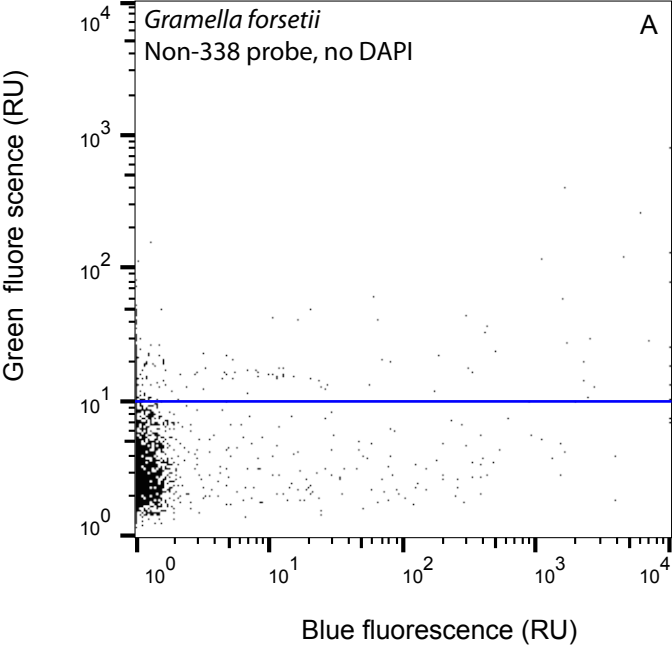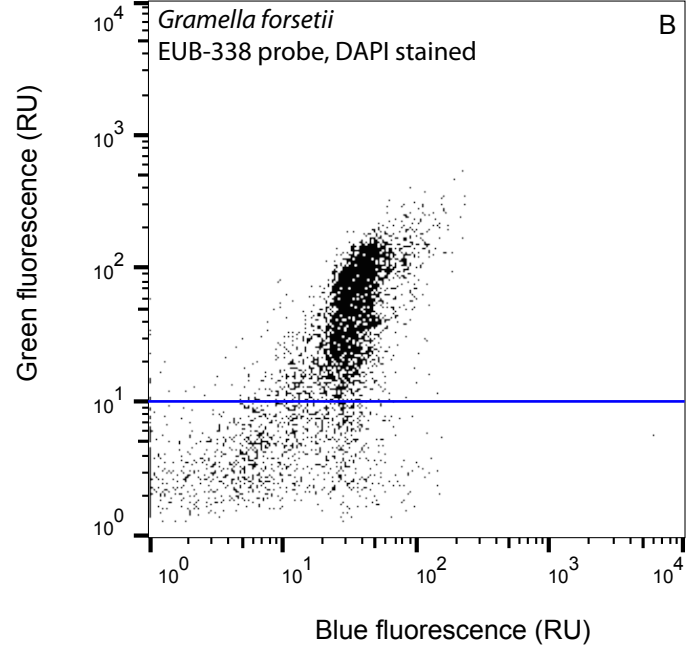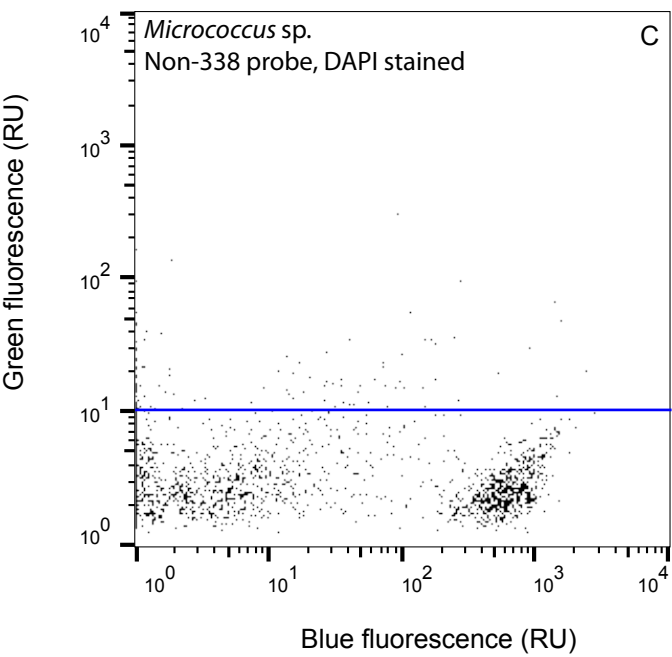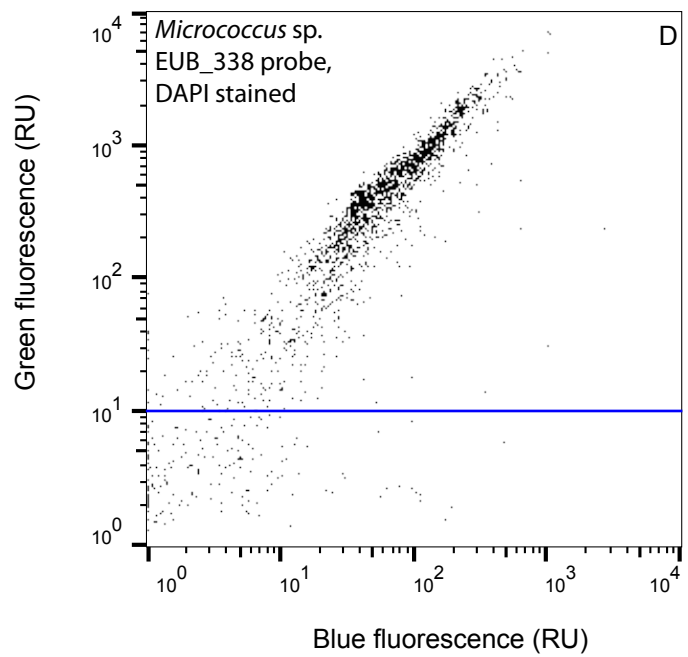

Supplement: Supplementary file 3 — Additional file 2: Figure S2. The background fluorescence in the green channel of flow cytometric measurements was set to 10 RU (blue line), based on comparisons betwen EUB-338 and Non-338 probes hybridized to 1% formaldehyde fixed Gramella forsetii (A, B) and Micrococcus sp. (C, D) samples. The Non-338 control of Gramella forsetii (A) was not stained with DAPI, the Non-338 control of Micrococcus sp. (C) was stained with DAPI. Green fluorescence was detected with a 530/40 nm filter, blue fluorescence with a 460/50 nm filter. 5000 events were recorded for Gramella forsetii (A, B) and 2000 for Micrococcus sp. (C, D). [file 40168_2020_790_MOESM2_ESM.pdf]

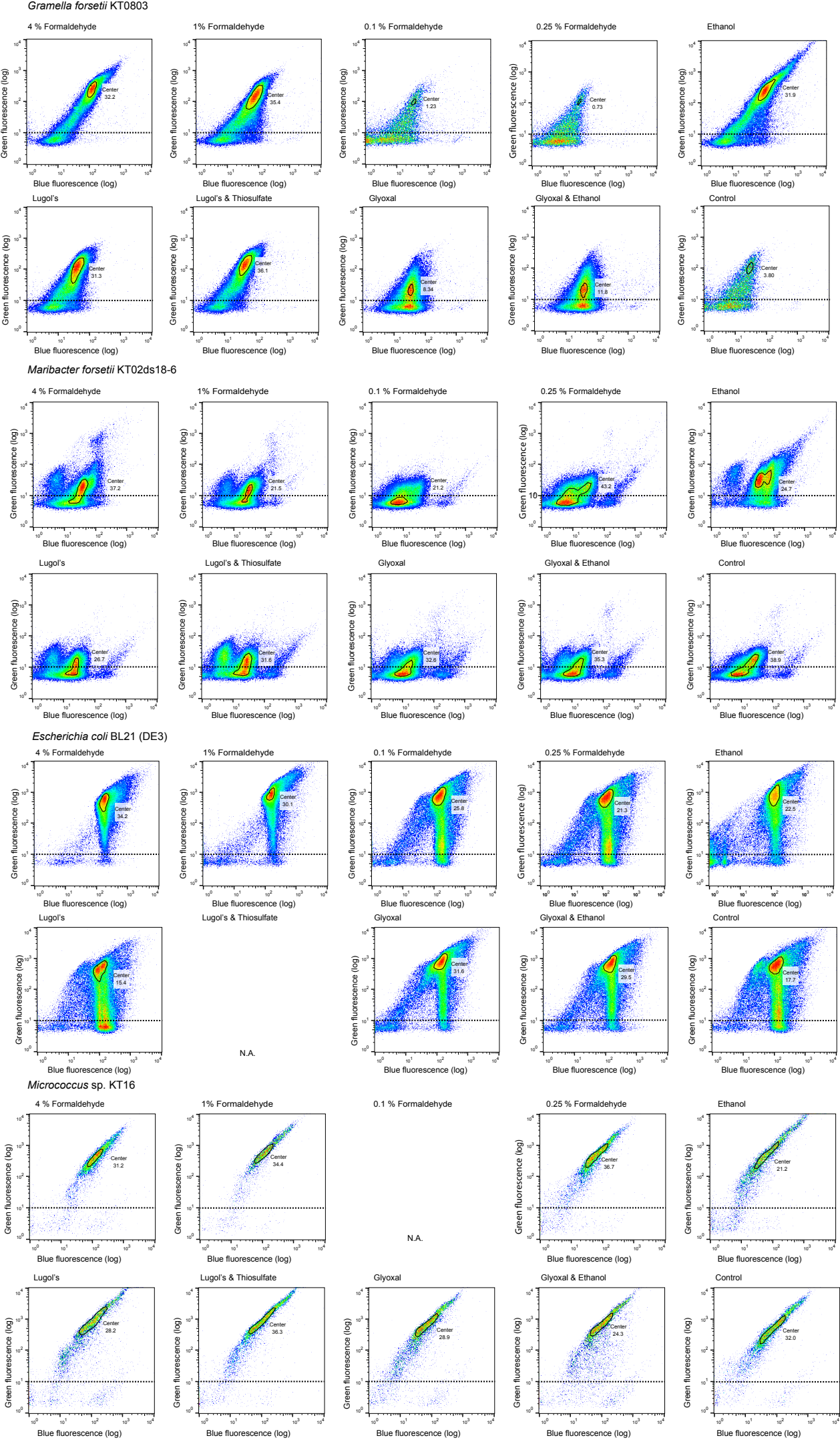

Supplement: Supplementary file 4 — Additional file 3: Figure S3. Four pure cultures (Gramella forsetii, Maribacter forsetii, Escherichia coli, Micrococcus sp.) were fixed with 10 different fixation methods (formaldehyde 4%, 1%, 0.25%, 0.1%, ethanol, Lugol’s solution with and without thiosulfate, glyoxal with and without ethanol and unfixed). HCR-FISH was done on filtered cells and signal intensity was measured after washing the cells off the filter and analyzing them in the flow cytometer. Plotted are the green fluorescence (530/40 nm) from HCR-FISH and blue fluorescence (450/60 nm) from DAPI staining. The fluorescence intensity is given in relative units on a logarithmic scale. The background fluorescence (dotted line) was defined for 10 RU. N.A. = not analyzed due to disrupted cells. [file 40168_2020_790_MOESM3_ESM.pdf]

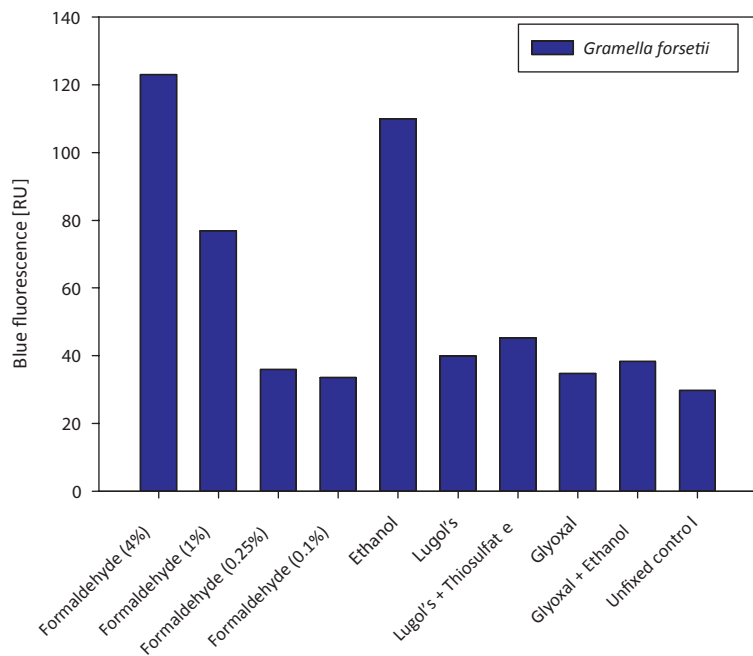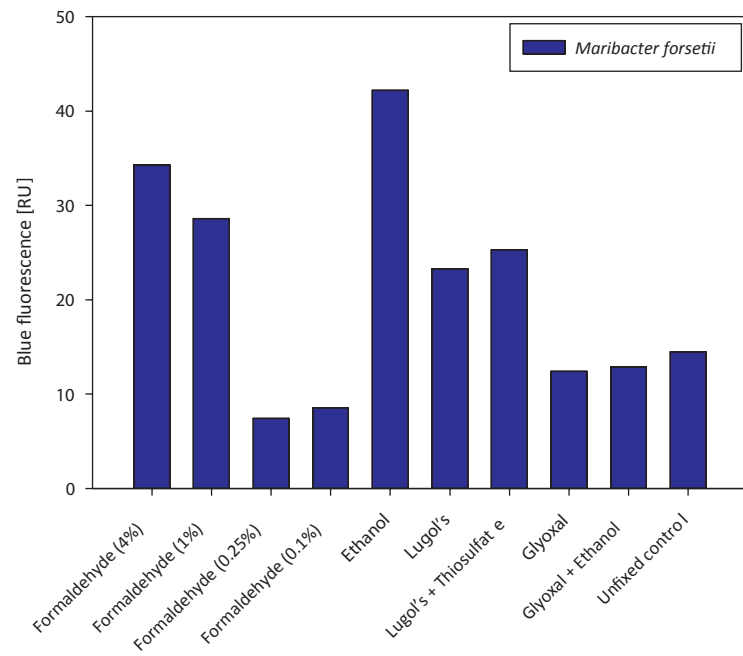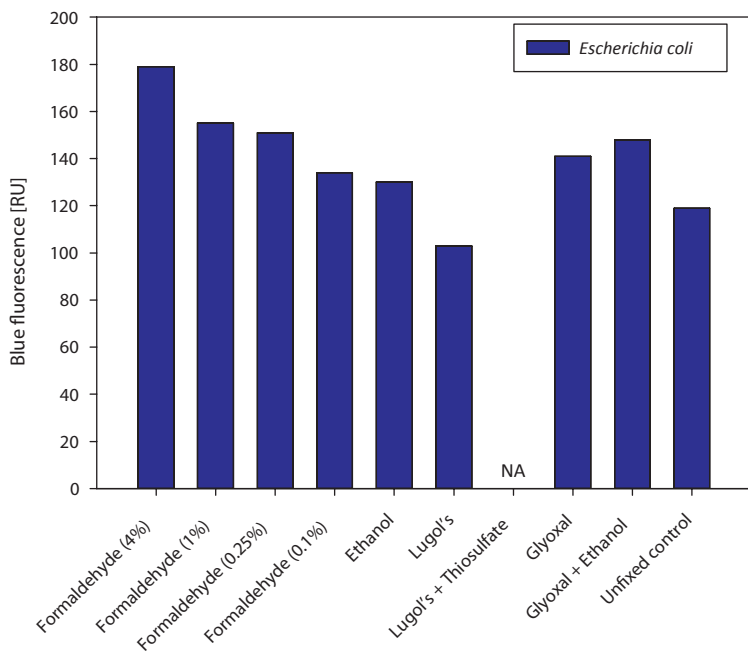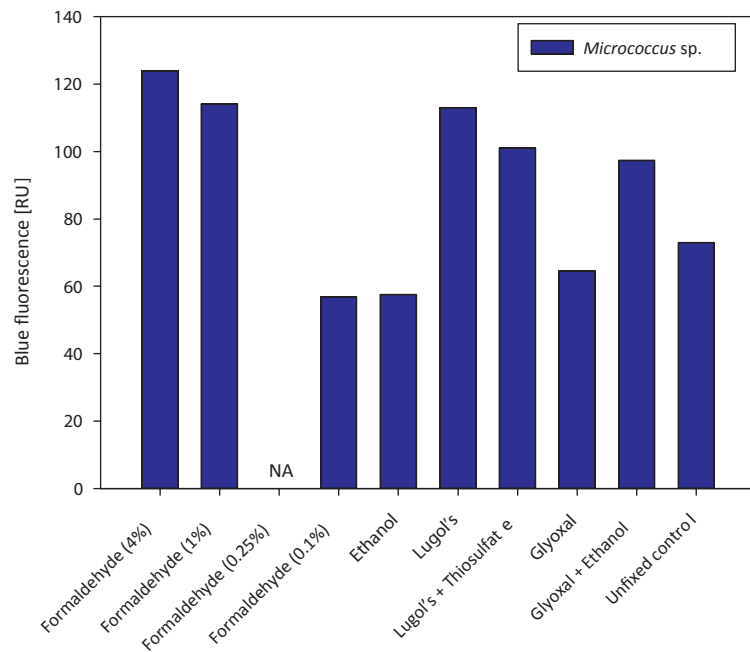

Supplement: Supplementary file 5 — Additional file 4: Figure S4. Blue fluorescence intensity (DAPI signal, 450/60 nm band-pass filter) of four isolates in dependency of cell fixation, measured by flow cytometry. The median of the signal population from flow cytometric analysis is shown. NA = not analyzed due to disrupted cells. [file 40168_2020_790_MOESM4_ESM.pdf]

***Gramella forsetii* (500 cells)**

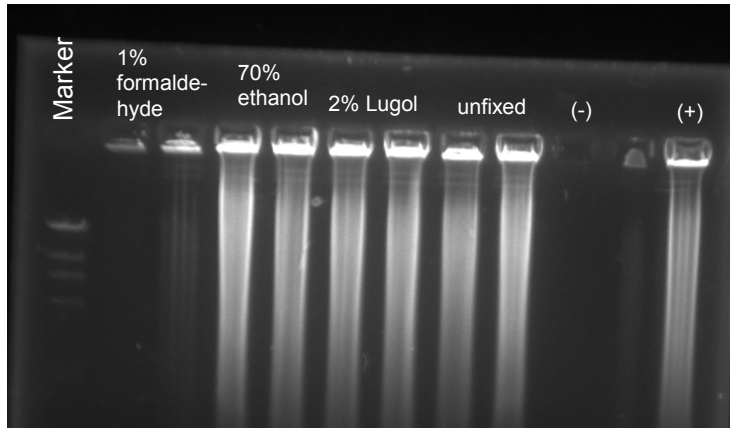

***Escherichia coli* (1000 cells)**

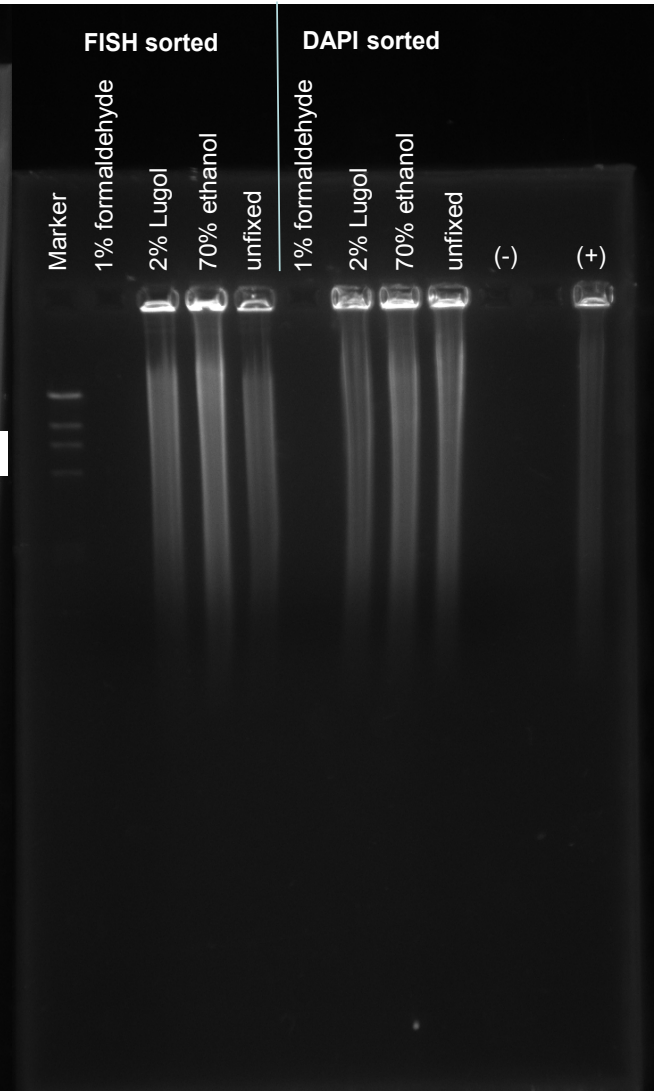

***Maribacter forsetii* (500 cells)**

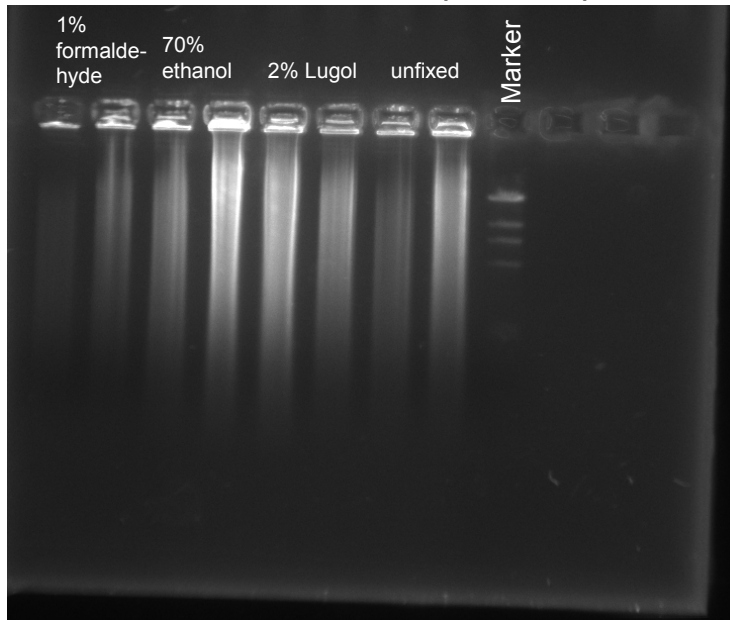

Supplement: Supplementary file 7 — Additional file 6: Figure S6. Exemplary images of gel electrophoresis with MDA products from sorted isolates, fixed with formaldehyde, Lugol’s solution, ethanol or unfixed. The uppermost band of the used marker LambdaDNA Hind III corresponds to 23 kb. MDA products from formaldehyde fixed cells were either not detectable or in low amount. [file 40168_2020_790_MOESM6_ESM.pdf]

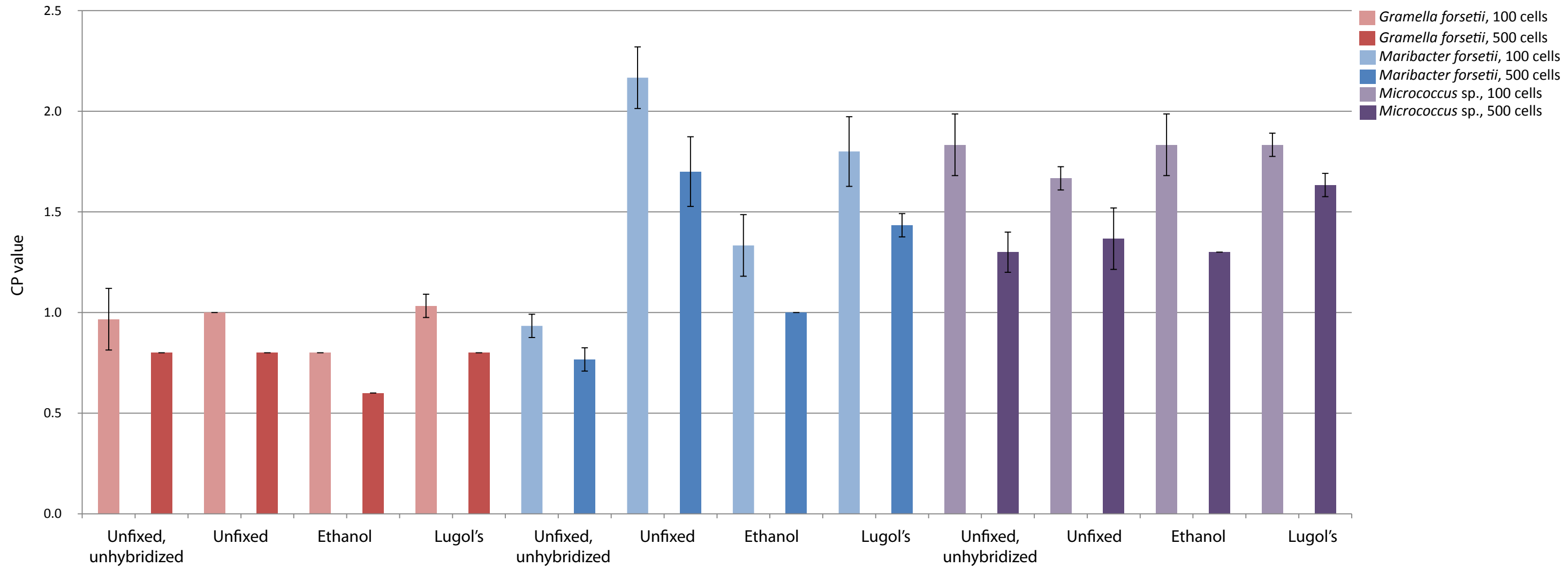

Supplement: Supplementary file 8 — Additional file 7: Figure S7. Crossing point times (CP, time of inflection point of real time amplification curve) of MDA reactions of three isolates with 100 or 500 cells input. Input samples were taken from Lugol’s fixed, ethanol fixed and unfixed cells. Additionally, unfixed cells that have not been subjected to FISH were used. [file 40168_2020_790_MOESM7_ESM.pdf]

## *Gramella forsetii*

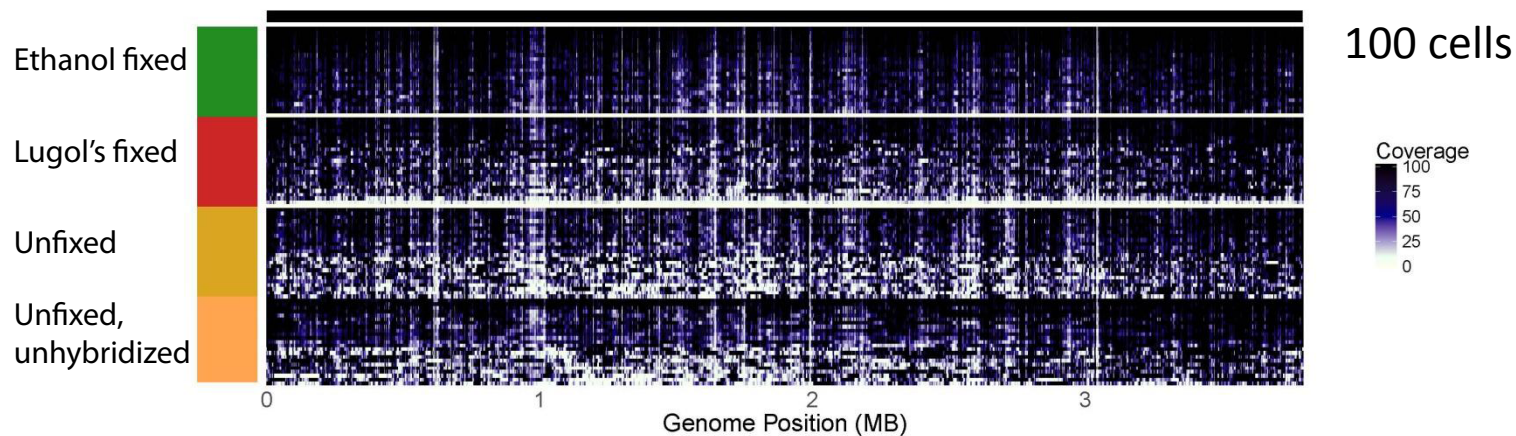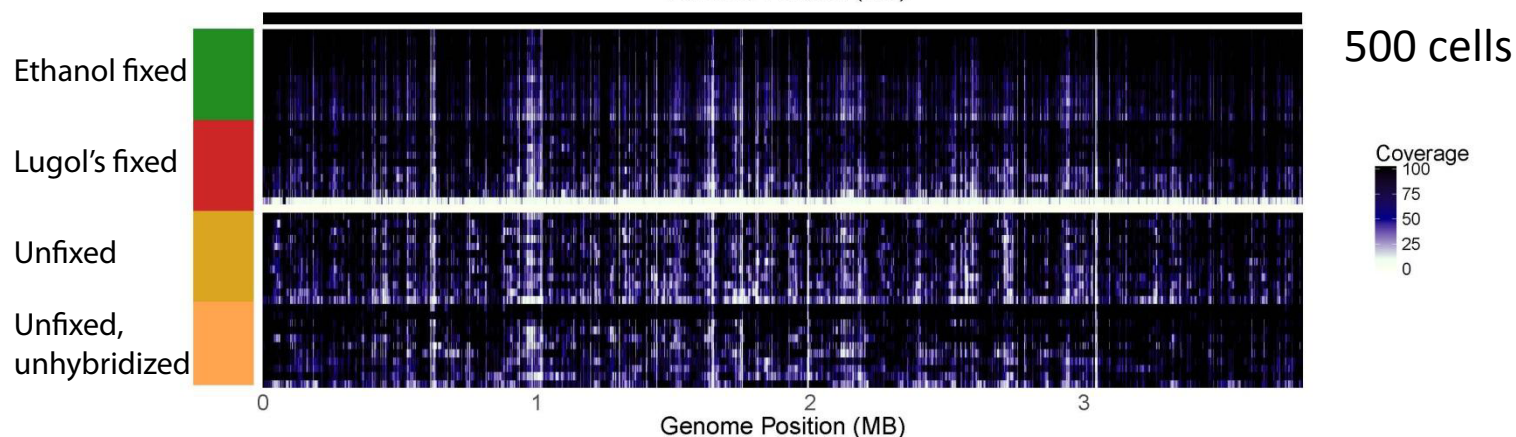

## *Maribacter forsetii*

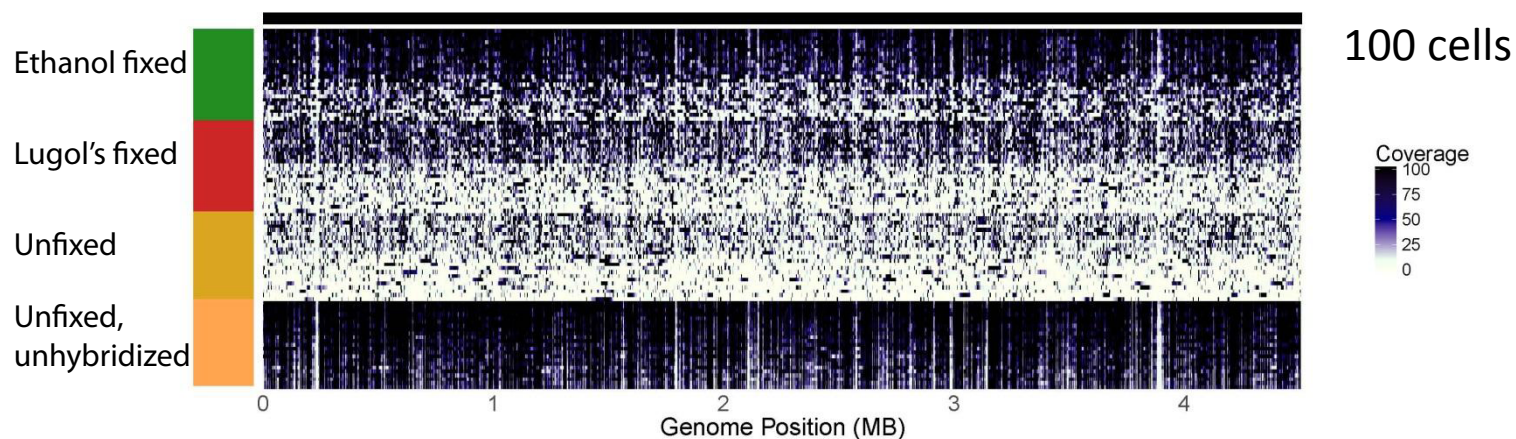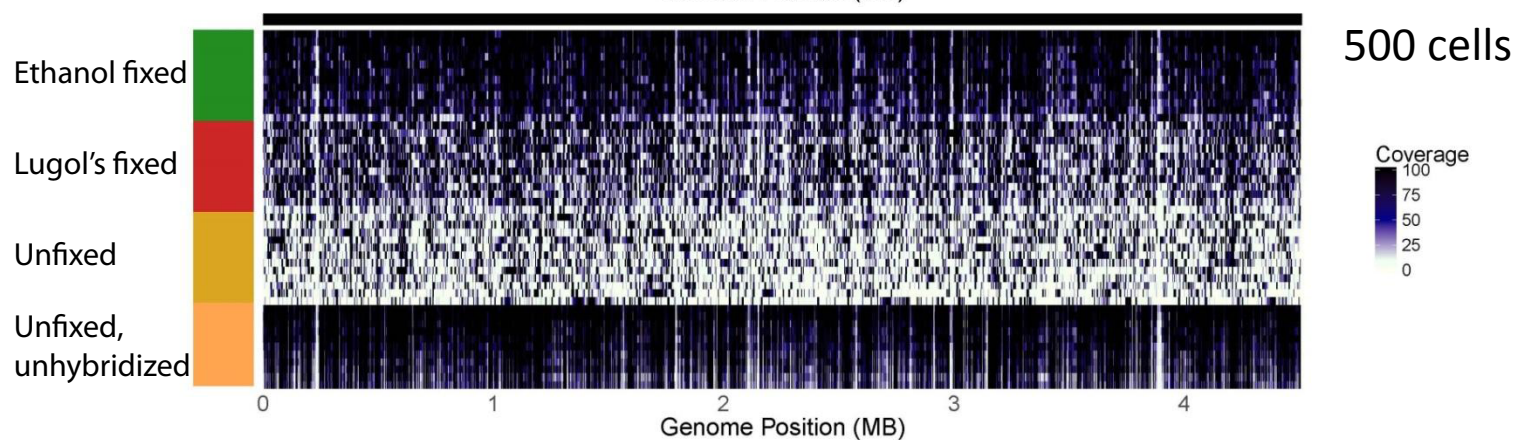

Supplement: Supplementary file 10 — Additional file 9: Figure S9. Read coverages across the reference genome of Gramella forsetii and Maribacter forsetii cells. 100 and 500 cells were sorted from isolates, used as input for MDA and the products were sequenced. Unfixed, unhybridized cells of Maribacter forsetii were taken directly from the culture without being filtrated and washed off the filter, which explains the difference in coverage between the treatments that we did not see for Gramella forsetii. [file 40168_2020_790_MOESM9_ESM.pdf]

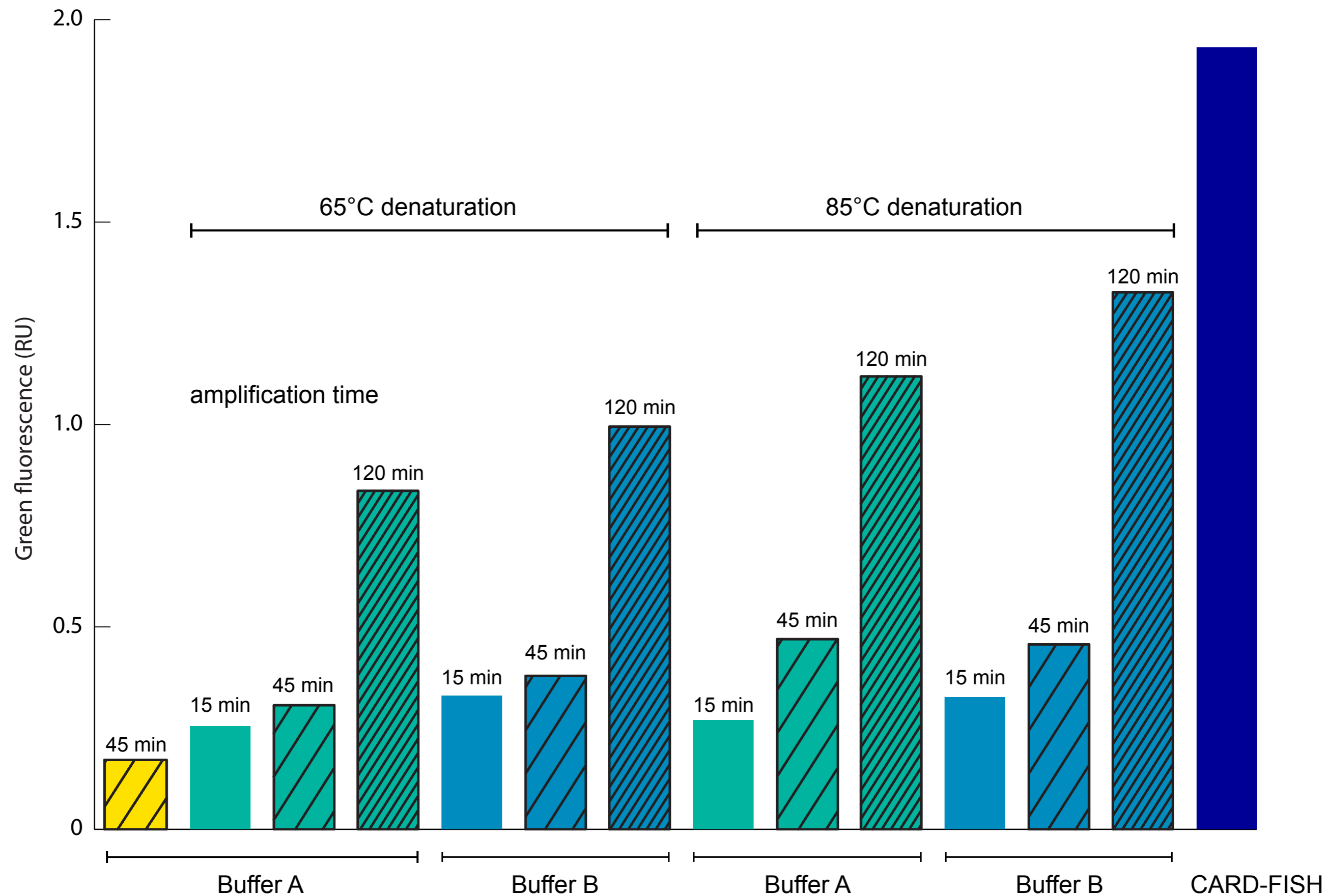

Supplement: Supplementary file 11 — Additional file 10: Figure S10. Signal intensity of formaldehyde fixed Gramella forsetii cells, after HCR-FISH with different treatments. CARD-FISH and the former protocol (yellow bar: in hybridization buffer A, no denaturation, 45 min amplification) were used for comparison. 30 min denaturation at 65°C and 85°C were tested in combination with hybridization buffer A and hybridization buffer B for three amplification times: 15 min, 45 min and 120 min. The signal intensities were measured via microscopy and are given in RU. The choice of hybridization buffer did not make a significant difference to signal intensity, but HCR-FISH signals were slightly higher when buffer B was used (see materials and methods for details in composition). Increasing chain reaction amplification time from 15 or 45 min to 120 min enhanced fluorescence from 0.3 (15 min) and 0.4 RU (45 min) to 1.1 RU (average values). [file 40168_2020_790_MOESM10_ESM.pdf]

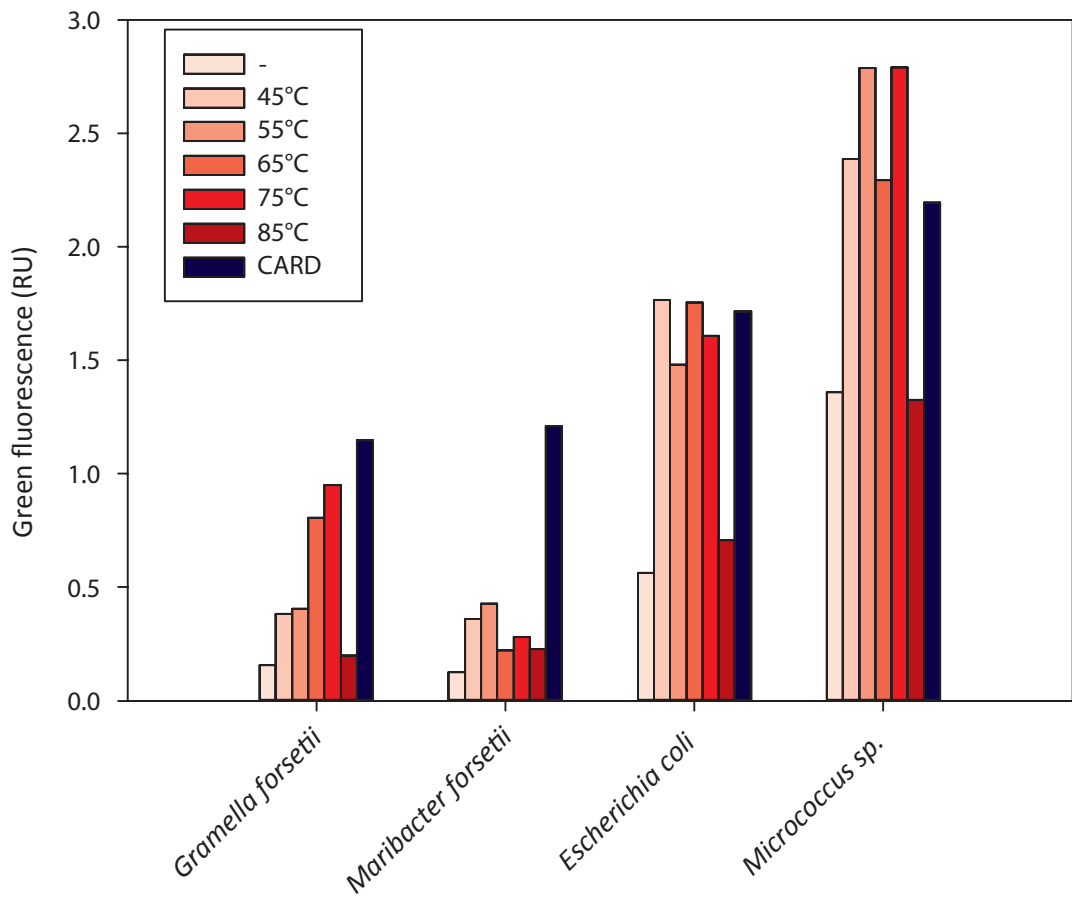

Supplement: Supplementary file 12 — Additional file 11: Figure S11. Signal intensity of four ethanol fixed isolates after HCR-FISH with 30 min denaturation (45-85°C) or without denaturation (-) and 2 h hybridization in comparison to CARD-FISH (CARD). Signal intensities were measured via microscopy and are given in RU. [file 40168_2020_790_MOESM11_ESM.pdf]

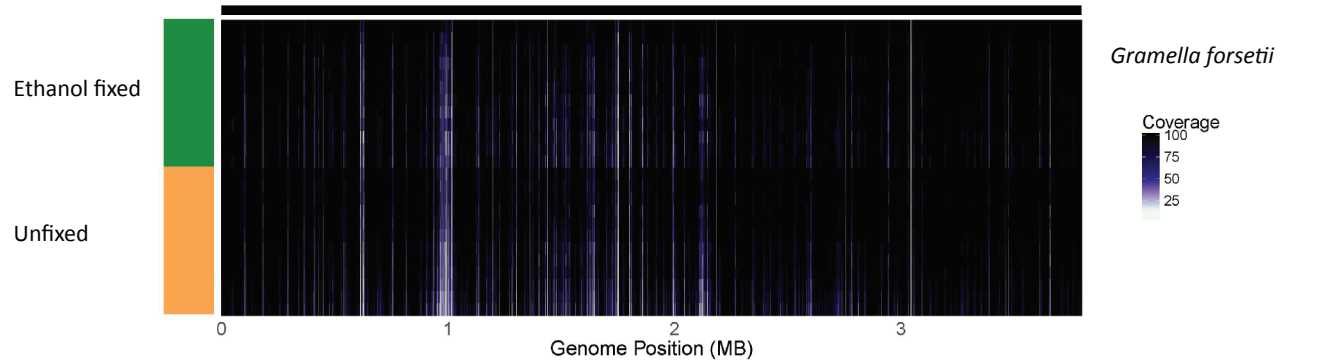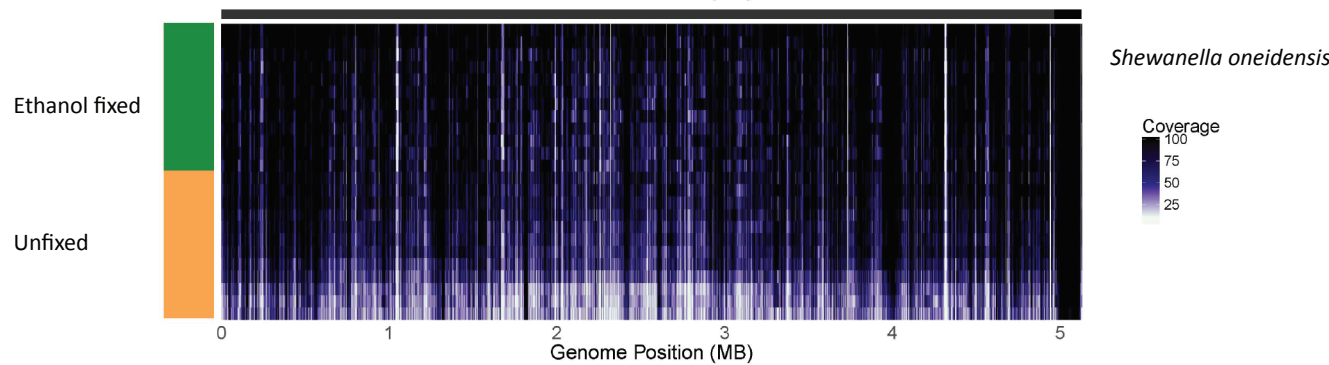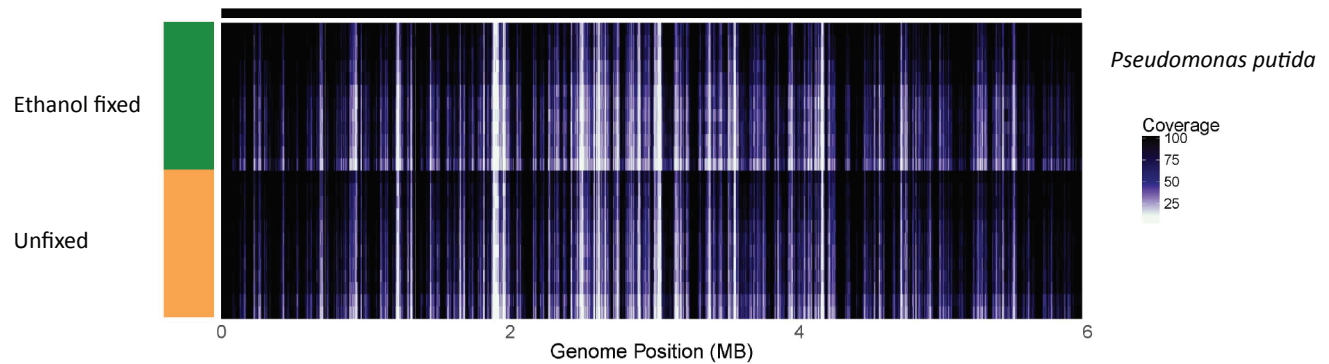

Supplement: Supplementary file 13 — Additional file 12: Figure S12. Sequencing read coverages across the reference genome of Gramella forsetii, Shewanella oneidensis and Pseudomonas putida. 500 cells from ethanol fixed (green) and unfixed (brown) samples were used as input for MDA. [file 40168_2020_790_MOESM12_ESM.pdf]

# A

## CF319a - *Bacteroidetes*

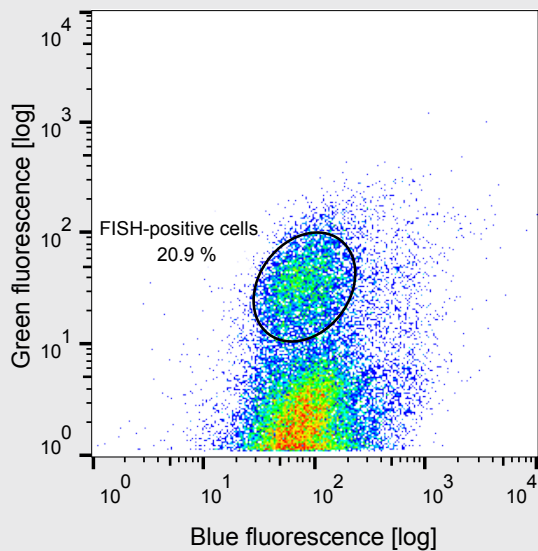

# B

## Vis6-814/871 - Vis6-clade

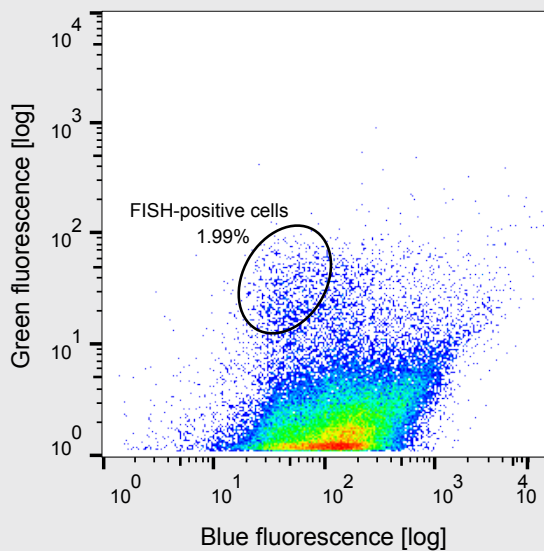

## Non338 - not binding

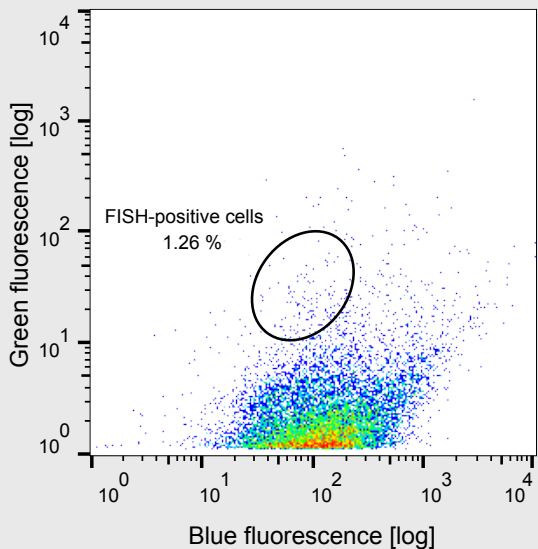

## Non338 - not binding

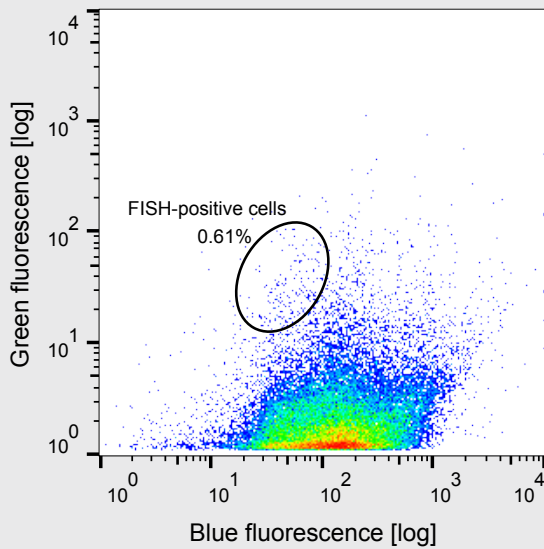

Supplement: Supplementary file 14 — Additional file 13: Figure S13. Flow cytometric sort gates of unfixed samples, targeted with A: CF319a (Bacteroidetes) probe and Non338 (not binding) probe and B: Targeted with the probe mix Vis6-814/871 (Vis6-clade) and Non338 probe. The blue fluorescence (355 nm laser, 450/60 nm detector) shows all bacteria stained with DAPI. The percentage indicates the number of detected events within the sort gate. The amount of signals with green fluorescence (488 nm laser, 530/40 nm detector) increase with samples targeted with a probe compared to the Non338 probe. [file 40168_2020_790_MOESM13_ESM.pdf]

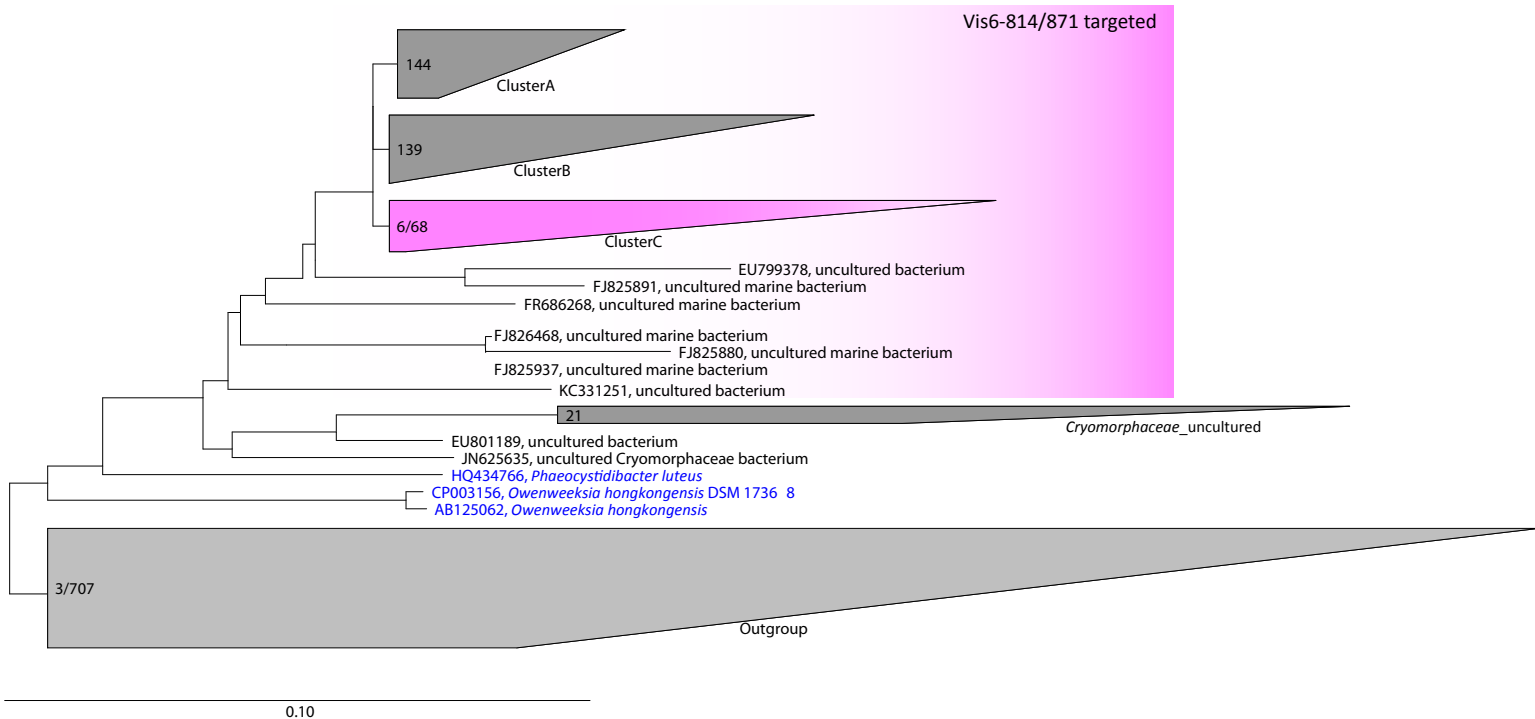

Supplement: Supplementary file 20 — Additional file 19: Figure S14. 16S rRNA consensus tree. The Vis6 cluster is targeted by the probes Vis6-814 and Vis6-871 and marked by the colored box. The closest cultured relative was Phaeocystitibacter luteus with appr. 90% sequence similarity. Nine 16S rRNA sequences, that have been binned to Vis6 MAGs from the Vis6 sorts, were placed in this tree. Six sequences were affiliated to Vis6 cluster C (marked in pink) and three sequences were affiliated to Flavobacterium ponti (in the outgroup). Flavobacterium ponti has appr. 86% 16S rRNA sequence identity to the Vis6 cluster. [file 40168_2020_790_MOESM19_ESM.pdf]

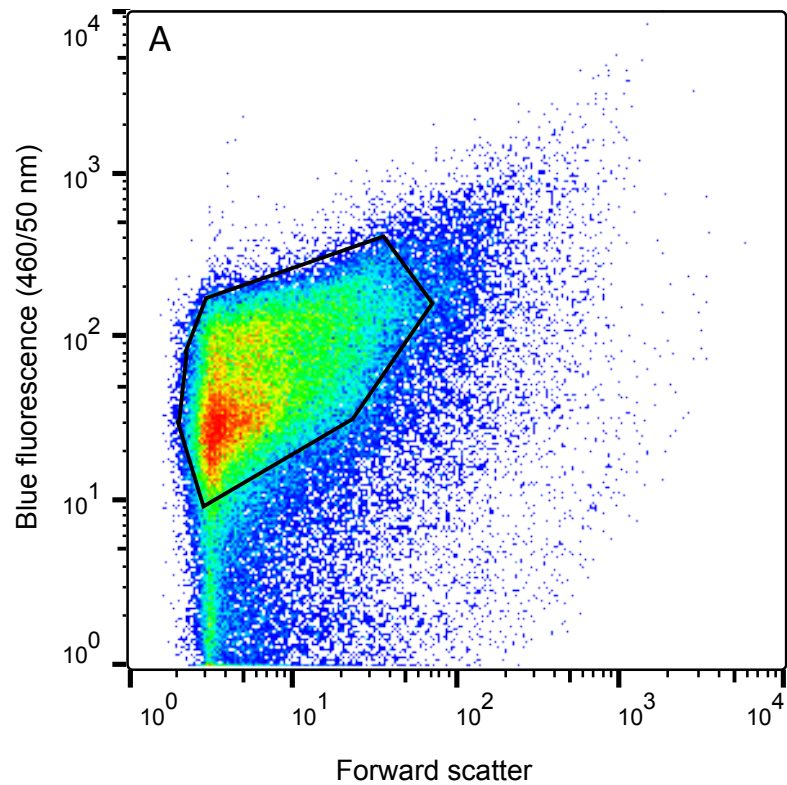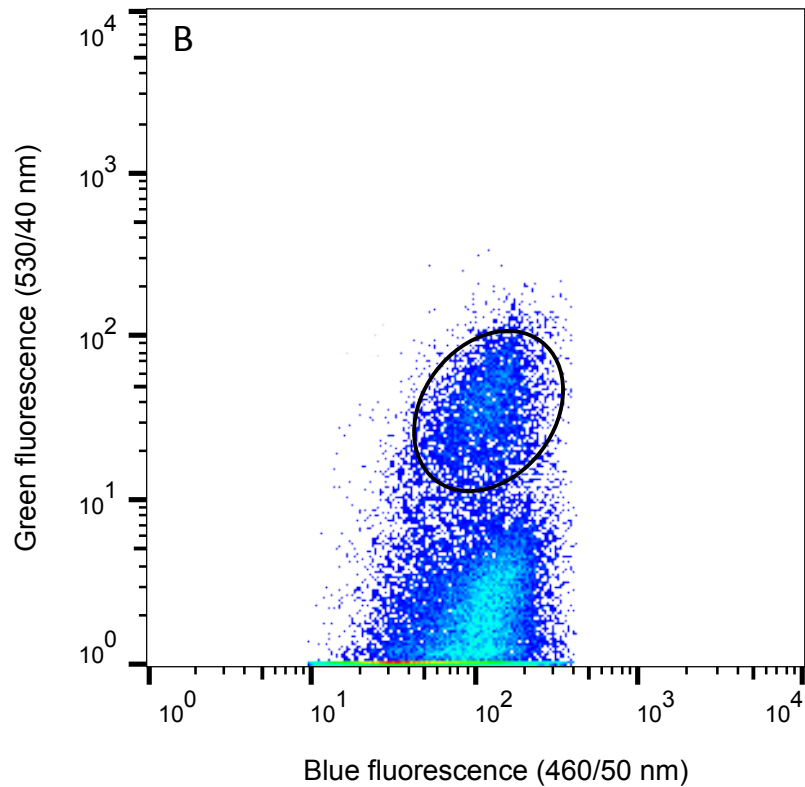

Supplement: Supplementary file 27 — Additional file 26: Figure S15. Exemplary scatter plots from flow cytometry showing the gating principle of seawater samples hybridized with HCR-FISH probe. (A) DAPI positive cells were selected in the forward scatter vs. blue fluorescence (DAPI signal) plot and (B) HCR-FISH positive cells were selected in the blue vs. green fluorescence (HCR-FISH signal) plot. Of (B) only those events that were also appearing in the gate in (A) were sorted. [file 40168_2020_790_MOESM26_ESM.pdf]
