# Supplementary material for: A pipeline for targeted metagenomics of environmental bacteria
Source: Microbiome. 2020 Feb 15;8:21. doi: 10.1186/s40168-020-0790-7 (PMC7024552; doi:10.1186/s40168-020-0790-7)
Supplement: Supplementary file 6 — Additional file 5: Figure S5. Microscopic images of differently fixed Gramella forsetii cells after cell sorting. Shown are overlay images of the HCR-FISH signal (green) and DAPI signal (blue). All images were taken with an epifluorescence microscope with a HC409LP (DAPI) and an ET500/LP (HCR-FISH) filter. The numbers in brackets indicate the exposure time for each image with the first number corresponding to the DAPI and the second to the HCR-FISH signals. [file 40168_2020_790_MOESM5_ESM.pdf]

.0.1 % Formaldehyde (60 / 250) | 0.25 % Formaldehyde (60 / 200) | 4 % Formaldehyde (130 / 250) | 1 % Formaldehyde (60 / 200)

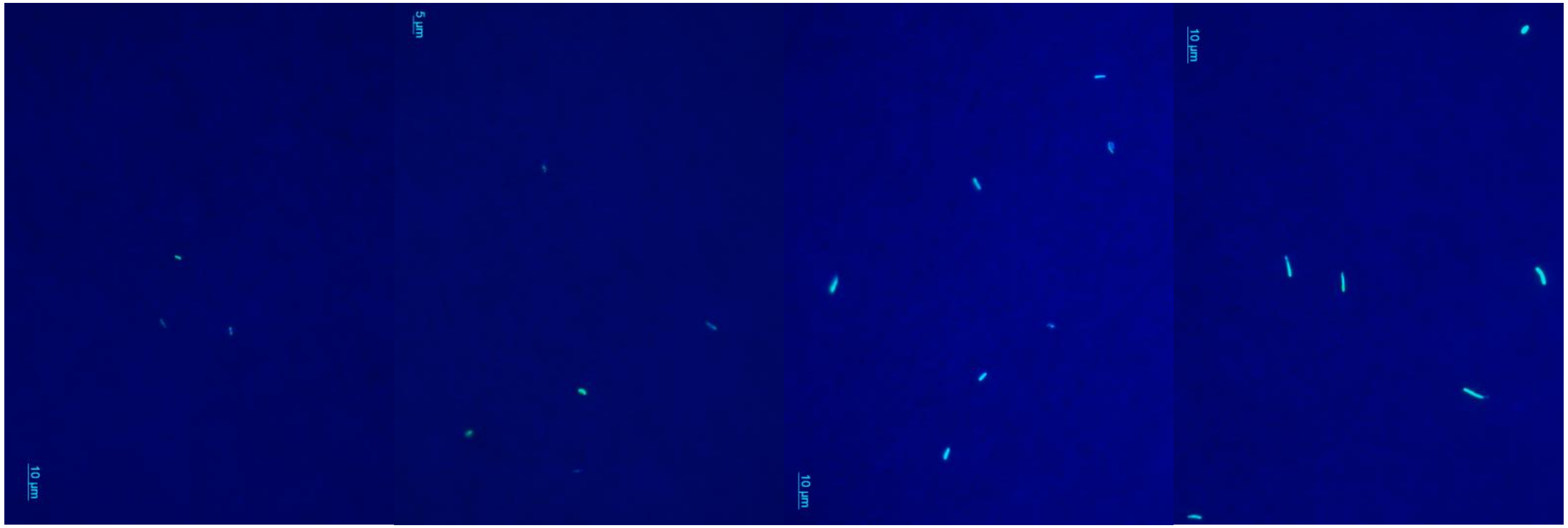

Glyoxal + Ethanol (60 / 300) | Glyoxal (60 / 250) | Lugol’s + Sodium thiosulfate (60 / 520) | Lugol’s solution (60 / 200)

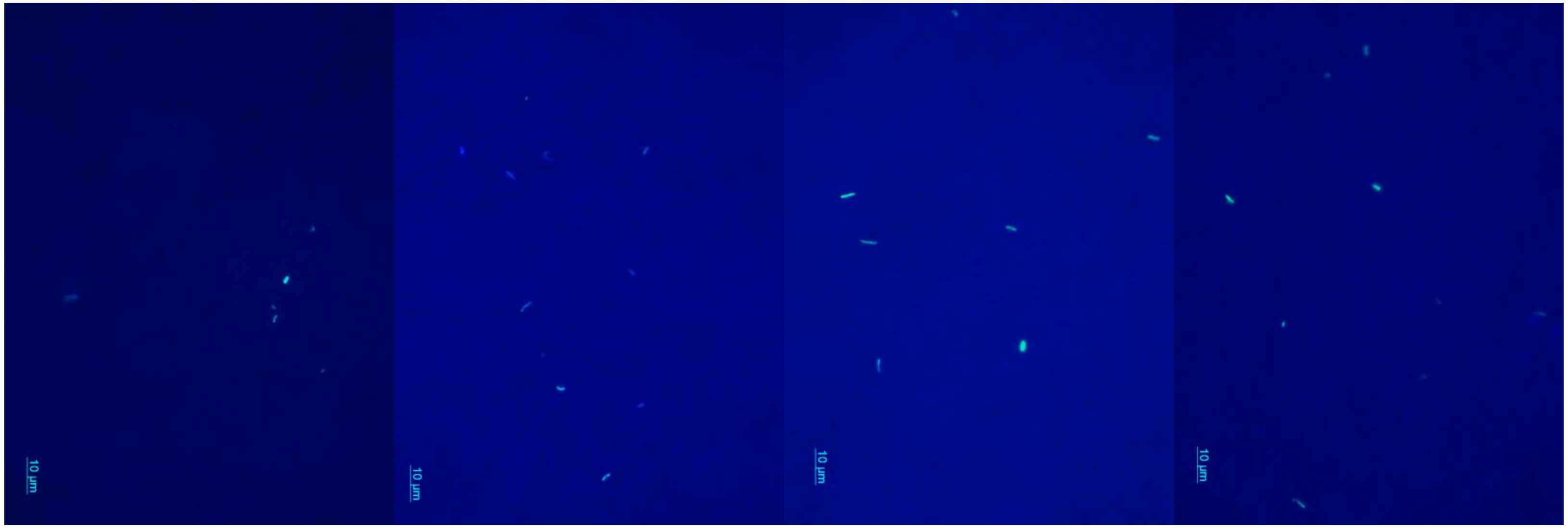

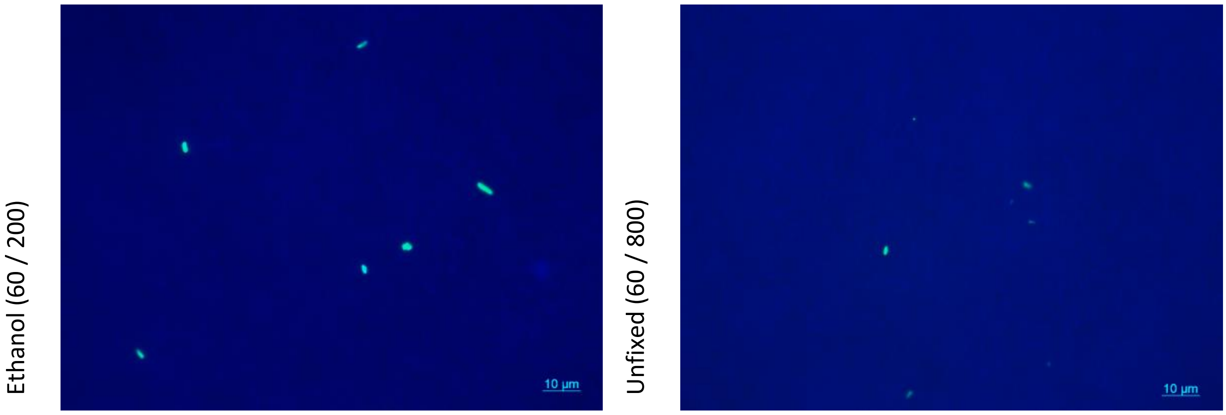
Ethanol (60 / 200)
10 µm
Unfixed (60 / 800)
10 µm
