# Supplementary material for: A pipeline for targeted metagenomics of environmental bacteria
Source: Microbiome. 2020 Feb 15;8:21. doi: 10.1186/s40168-020-0790-7 (PMC7024552; doi:10.1186/s40168-020-0790-7)
Supplement: Supplementary file 9 — Additional file 8: Figure S8. Genomic quality estimation of Gramella forsetii and Maribacter forsetii sequencing products after MDA of 100 and 500 sorted cells using Quast. Shown are from left to right: the number of contigs longer than 1000 bp, the total assembly lengths, N50, number of misassemblies and number of mismatches per 100 kpb. Unhybridized samples were unfixed and were not subjected to HCR-FISH, but only sorted based on their DAPI signal. Maribacter forsetii unhybridized controls were taken from a cell aliquot and have not been filtered and washed off a filter like the other controls, including the Gramella forsetii unhybridized control. Significance thresholds (p-values) of pairwise t-tests are * < 0.05, ** < 0.01, *** < 0.001. [file 40168_2020_790_MOESM8_ESM.pdf]

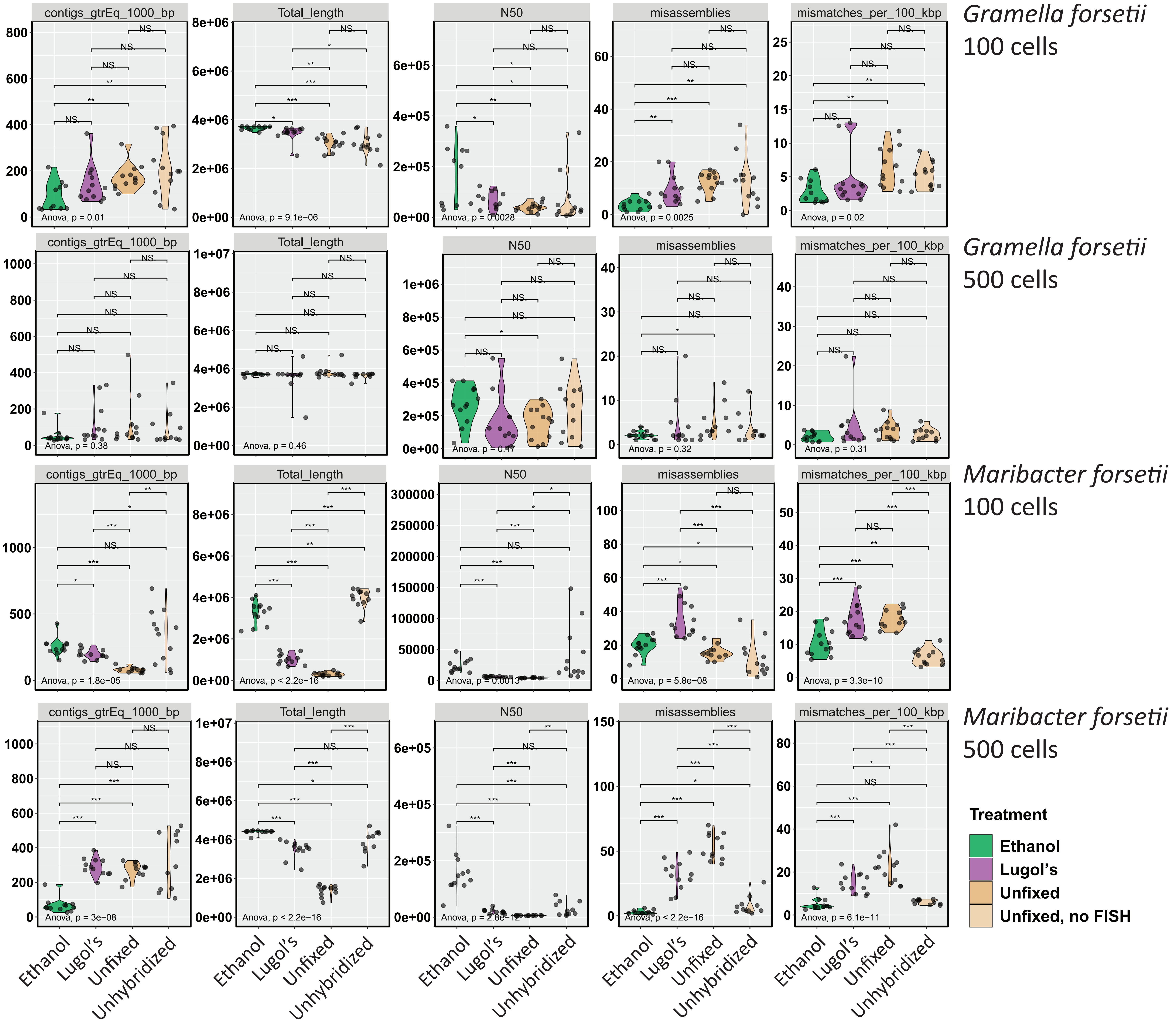
Gramella forsetii
100 cells
Gramella forsetii
500 cells
Maribacter forsetii
100 cells
Maribacter forsetii
500 cells
contigs_gtrEq_1000_bp
Total_length
N50
misassemblies
mismatches_per_100_kbp
Anova, p = 0.01
Anova, p = 9.1e−06
Anova, p = 0.0028
Anova, p = 0.0025
Anova, p = 0.02
Anova, p = 0.38
Anova, p = 0.46
Anova, p = 0.17
Anova, p = 0.32
Anova, p = 0.31
Anova, p = 1.8e−05
Anova, p < 2.2e−16
Anova, p = 0.0013
Anova, p = 5.8e−08
Anova, p = 3.3e−10
Anova, p = 3e−08
Anova, p < 2.2e−16
Anova, p = 2.8e−12
Anova, p < 2.2e−16
Anova, p = 6.1e−11
Ethanol
Lugol's
Unfixed
Unhybridized
Treatment
Ethanol
Lugol's
Unfixed
Unfixed, no FISH
