# Supplementary material for: A pipeline for targeted metagenomics of environmental bacteria
Source: Microbiome. 2020 Feb 15;8:21. doi: 10.1186/s40168-020-0790-7 (PMC7024552; doi:10.1186/s40168-020-0790-7)
Supplement: Supplementary file 25 — Additional file 24: Table S10. Bacterial strains used for benchmarking the influence of cell fixation and for HCR-FISH optimization. [file 40168_2020_790_MOESM24_ESM.docx]

Table S10: Bacterial strains used for benchmarking the influence of cell fixation and for HCR-FISH optimization. ^*^RT: room temperature (20-25°C) ^**^SY medium: 40 g sea salts, 1 g yeast extract / L ^***^LB medium: 5 g yeast extract, 10 g tryptone, 5 g sodium chloride / L ^****^Micrococcus sp. KT16 was grown in SY medium for the fixation experiment and in LB medium for the HCR-FISH optimization by microscopy. ^*****^BD, cat 234000

| Strain | Family | GC-content (mol%) | Reference | Genome Acc. No. NCBI | Growth conditions |
| --- | --- | --- | --- | --- | --- |
| *Gramella forsetii* KT0803 | *Flavobacteriaceae* | 36.6 | [Bauer *et al.* (2006](#_ENREF_2)) | CU207366 | RT^*^, SY medium^**^ |
| *Maribacter forsetii* KT02ds18-6 | *Flavobacteriaceae* | 34.2-34.4 | [Barbeyron *et al.* (2008](#_ENREF_1)) | NZ_JQLH01000000 | RT, SY medium |
| *Escherichia coli* BL21 (DE3) | *Enterobacteriaceae* | 50.6 | Sigma-Aldrich, CMC0014 | CP001509 | 37°C, LB medium^***^ |
| *Micrococcus* sp. KT16 | *Micrococcaceae* | 73.0 | IMG GOLD ID: Ga0256418 | ASM331501v1 | RT, SY medium/LB medium^****^ |
| *Pseudomonas putida* F1 | *Pseudomonadaceae* | 62.2 | [Finette *et al.* (1984](#_ENREF_3)) | NC_002947 | 30°C, nutrient broth^*****^ |
| *Shewanella oneidensis* MR-1 | *Shewanellaceae* | 46.0 | [Venkateswaran *et al.* (1999](#_ENREF_4)) | NC_004347 | 30°C, LB medium |

Barbeyron, T., Carpentier, F., L'Haridon, S., Schuler, M., Michel, G. and Amann, R. (2008). "Description of *Maribacter forsetii* sp nov., a marine *Flavobacteriaceae* isolated from North Sea water, and emended description of the genus *Maribacter*." International Journal of Systematic and Evolutionary Microbiology **58**: 790-797.

Bauer, M., Kube, M., Teeling, H., Richter, M., Lombardot, T., Allers, E., Würdemann, C. A., Quast, C., Kuhl, H., Knaust, F., Woebken, D., Bischof, K., Mussmann, M., Choudhuri, J. V., Meyer, F., Reinhardt, R., Amann, R. I. and Glöckner, F. O. (2006). "Whole genome analysis of the marine *Bacteroidetes* ‘*Gramella forsetii*’ reveals adaptations to degradation of polymeric organic matter." Environmental Microbiology **8**(12): 2201-2213.

Finette, B. A., Subramanian, V. and Gibson, D. T. (1984). "Isolation and characterization of Pseudomonas putida PpF1 mutants defective in the toluene dioxygenase enzyme system." Journal of Bacteriology **160**(3): 1003-1009.

Venkateswaran, K., Moser, D. P., Dollhopf, M. E., Lies, D. P., Saffarini, D. A., MacGregor, B. J., Ringelberg, D. B., White, D. C., Nishijima, M., Sano, H., Burghardt, J., Stackebrandt, E. and Nealson, K. H. (1999). "Polyphasic taxonomy of the genus *Shewanella* and description of *Shewanella oneidensis* sp. nov." International Journal of Systematic and Evolutionary Microbiology **49**(2): 705-724.
