# Supplementary material for: A pipeline for targeted metagenomics of environmental bacteria
Source: Microbiome. 2020 Feb 15;8:21. doi: 10.1186/s40168-020-0790-7 (PMC7024552; doi:10.1186/s40168-020-0790-7)
Supplement: Supplementary file 26 — Additional file 25: Table S11. Probes used in FISH experiments. In HCR-FISH, the probe consists of a taxon specific sequence attached to the initiator sequence. The G’s in bold of the hairpin oligonucleotides are labelled with Atto488 (Biomers). [file 40168_2020_790_MOESM25_ESM.docx]

Table S11: Probes used in FISH experiments. In HCR-FISH, the probe consists of a taxon specific sequence attached to the initiator sequence. The G’s in bold of the hairpin oligonucleotides are labelled with Atto488 (Biomers). *Formamide concentrations of helper and competitor oligos are adapted to the concentration of the probe ** The CF319a probe targets appr. 50% of Bacteroidetes, including the majority of Flavobacteriales and Sphingobacteriales which are the major clades of marine Bacteroidetes.

| **Probe** | **Specificity** | | **Sequence (5’-3’)** | **Formamide [%]** | **Reference** |
| --- | --- | --- | --- | --- | --- |
| Eub-338-1 | Bacteria | | GCTGCCTCCCGTAGGAGT | 35 | Amann et al. (1990) |
| Non-338 | No target | | ACTCCTACGGGAGGCAGC | 35 | Wallner et al. (1993) |
| CF319a | Bacteroidetes ** | | TGGTCCGTGTCTCAGTAC | 35 | Manz et al. (1996) |
| Vis6-814 | Vis6-clade | | CAGCGAGTGATGATCGTT | 15 | Gómez-Pereira et al. (2010) |
| Vis6-814_c | Competitor for Vis6-814 | | CAGCGAGTGATCATCGTT | * | Gómez-Pereira et al. (2010) |
| Vis6-814_h1 | Helper for Vis6-814 | | TACGGCGTGGACTACCAGGT | * | Hahnke et al. (2015) |
| Vis6-814_h2 | Helper for Vis6-814 | | CCGCYGACAGTATATCGCCAA | * | Hahnke et al. (2015) |
| Vis6-871 | Vis6-clade | | CCCCAGGTGCATCACTTA | 15 | This study |
| **Probe** | | **Sequence (5’-3’)** | | | **Reference** |
| Initiator-sequence (5’-3’) | | CCGAATACAAAGCATCAACGACTAGAAAAAA-Probe | | | Yamaguchi et al. (2015) |
| Hairpin oligo H1 | | TCTAGTCGTT**G**ATGCTTT**G**TATTCGGCGACA**G**ATAACCGAATACAAA**G**CATC | | | Choi et al. (2010) |
| Hairpin oligo H2 | | CCGAATACAAA**G**CATCAAC**G**ACTAGAGATGCTTT**G**TATTCG**G**TTATCTGTCG | | | Choi et al. (2010) |
